# Causal influences of migraine on neuropsychiatric disorders: A 2-sample Mendelian randomization study

**DOI:** 10.1097/MD.0000000000046041

**Published:** 2025-12-19

**Authors:** Tiantian Xu, Qinlong Gao, Lingsan Hu, Bing Deng, Qing Ren, Genfa Du, Dan Xi

**Affiliations:** a Shenzhen Bao’an Traditional Chinese Medicine Hospital, Guangzhou University of Chinese Medicine, Shenzhen, Guangdong, China; b Guangzhou University of Chinese Medicine, Guangzhou, Guangdong, China; c The First Clinical Medical College, Hainan Medical University, Haikou, Hainan, China.

**Keywords:** causality, Mendelian randomization, migraine, neuropsychiatric disorders

## Abstract

Our study aims to explore the causal relationship between migraine (with and without aura) and 6 disorders: anxiety, bipolar disorder, epilepsy, major depressive disorder, insomnia, and stroke, using a 2-sample Mendelian randomization. Migraine without aura had a significant causal association with bipolar disorder (odds ratios = 1.075, 95% confidence intervals = [1.005–1.151], *P* = .037). A complex relationship was found with major depressive disorder (odds ratios = 1.036, 95% confidence intervals = [1.009–1.064], *P* = .010 for overall migraine). But after correction for Benjamini–Hochberg multiple tests, no causal links were identified between migraine (with and without aura) and these 6 disorders (all *P*_FDR_ > .05). Further biological and clinical studies are needed to validate these findings.

## 1. Introduction

Migraine, a prevalent and disabling neurological disorder, is characterized by recurring episodes of severe headache often accompanied by symptoms such as nausea, photophobia, and phonophobia.^[1]^ It affects approximately 14% of the global population, with a higher prevalence in women than in men.^[2]^ The socioeconomic burden of migraine is substantial, significantly impacting quality of life and contributing to healthcare costs and productivity losses.^[3]^

Neuropsychiatric disorders such as depression, anxiety, bipolar disorder (BD), insomnia, stroke, and epilepsy are highly prevalent and contribute substantially to global disability and mortality.^[4]^ Among these, migraine is frequently comorbid with several psychiatric conditions, including major depression disorder (MDD), anxiety, BD, insomnia, stroke, and epilepsy.^[5]^ Growing evidence supports a complex relationship between migraine and these disorders, suggesting shared underlying mechanisms.^[5–8]^ Specially, migraine and MDD demonstrate a strong bidirectional association, with each disorder increasing the risk of the other. This comorbidity may be attributed to common genetic factors and dysregulation within hypothalamic and thalamic pathways. Individuals with migraine are 2 to 5 times more likely to develop depression, a risk that is especially elevated among women.^[9,10]^ Similarly, migraine shows a robust link with anxiety disorders, including generalized anxiety and panic disorder, with odds ratios (OR) typically ranging from 2 to 5. This bidirectional relationship may involve shared neurobiological abnormalities, including serotonergic dysfunction and hypothalamic–pituitary–adrenal axis dysregulation.^[11,12]^ Additionally, migraine and epilepsy co-occur at higher rates than expected, likely due to genetic susceptibility and neuronal hyperexcitability.^[13]^ Although migraine may elevate the risk of ischemic events that could subsequently lead to epilepsy or stroke, direct causal pathways remain under investigation.^[8]^ Furthermore, migraine is strongly associated with insomnia, where increased headache frequency and pain intensity correlate with a greater risk of sleep disturbances.^[14]^

Based on the foregoing analysis, we ascertain that migraine is closely linked to anxiety, depression, insomnia, epilepsy, BD, and stroke. Additionally, individuals with migraine are at an elevated risk of developing neuropsychiatric disorders, and conversely, those with neuropsychiatric disorders are more prone to experiencing migraines.^[9,15–17]^ However, the causal nature of these associations remains unclear due to potential confounding factors and the limitations of observational studies. For example, stressful life events associated with migraine may lead to depression,^[18]^ or preexisting depression may exacerbate migraine symptoms.^[19]^ Shared genetic and environmental risk factors could also contribute to the comorbidity of migraine and neuropsychiatric disorders, complicating the interpretation of these associations.

To address these limitations, a more robust methodological approach is needed to elucidate the causal relationship between migraine and neuropsychiatric disorders. Mendelian randomization (MR) is a genetic epidemiological technique that uses genetic markers as instrumental variables (IVs) to establish causal relationships between exposure and outcome.^[20]^ MR studies can mitigate confounding and reverse causation, providing more reliable evidence for causal inferences. While recent MR studies have investigated the causal links between neuropsychiatric disorders and various health outcomes,^[21,22]^ few have specifically addressed the causal impact of migraine on neuropsychiatric disorders. The objective of this study is to explore the potential causal relationship between migraine and neuropsychiatric disorders using a 2-sample MR approach. The findings may enhance our understanding of the complex interplay between these conditions and inform more effective clinical management and prevention strategies.

## 2. Materials and methods

### 2.1. Study design

**Figure 1.**
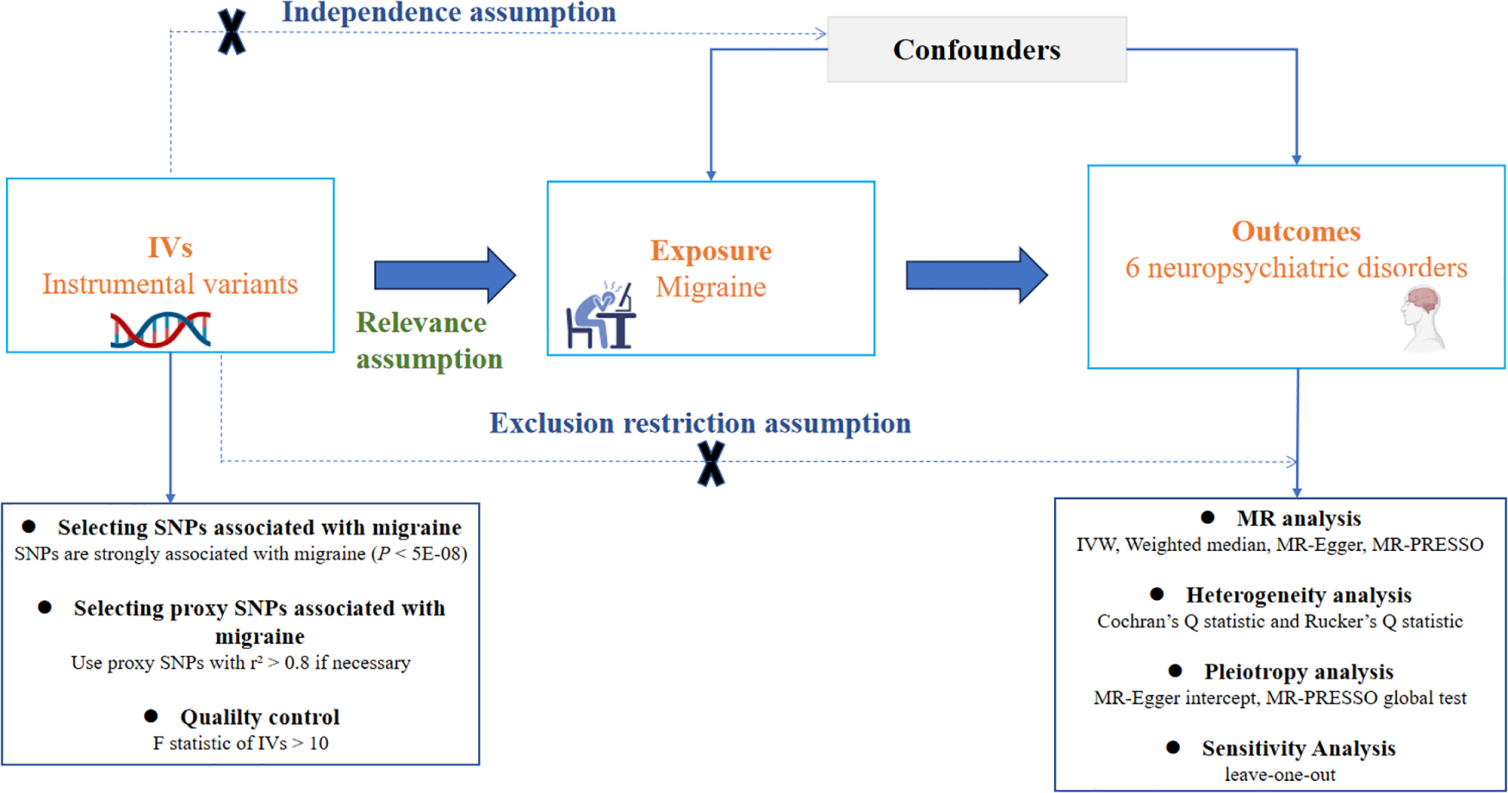
Design and main assumptions of Mendelian randomization study.

A 2-sample MR study was conducted to determine the causal relationship between migraine and 6 neuropsychiatric disorders, using genetic variations, such as single nucleotide polymorphisms (SNPs), as IVs.^[23]^ This method involves obtaining association estimates: summary statistics between genotype and exposure and genotype and outcome: from 2 distinct datasets with minimal individual overlap.^[24]^ Valid SNPs as IVs must satisfy 3 assumptions^[25]^: SNPs must be closely associated with exposure (relevance hypothesis); SNPs must influence the outcome only through exposure (exclusion restriction); and SNPs must be independent of confounders affecting the exposure–outcome relationship (independence assumption) (Fig. 1).

### 2.2. Data sources

We utilized recent genome-wide or whole-genome meta-analysis data from the Psychiatric Genomics Consortium available at https://www.med.unc.edu/pgc/download-results/. These datasets included anxiety disorder,^[26]^ BD,^[27]^ epilepsy,^[28]^ major depressive disorder (MDD),^[29]^ and insomnia.^[30]^ Psychiatric Genomics Consortium has made significant contributions to understanding the genetic architecture of neuropsychiatric disorders.^[31]^ GWAS summary data pertaining to stroke and its subtypes were acquired from the MEGASTROKE Consortium meta-analysis, which encompassed 438,847 individuals of European ancestry (40,585 cases and 406,111 controls). Stroke mainly results from cerebral infarction (ischemic stroke), with fewer cases due to intracerebral hemorrhage. Ischemic stroke is categorized into large atherosclerotic stroke (LAS), cardioembolic stroke (CES), and small vessel disease (SVS) stroke.

Migraine is classified into migraine with aura (MA) and migraine without aura (MO). We analyzed migraine (23,899 cases and 341,884 controls), MA (10,336 cases and 341,884 controls), and MO (8626 cases and 341,884 controls) from the GWAS database, with all data derived from the same study, FinnGen-R11.^[32]^ Please refer to the original publication for comprehensive sample descriptions, genotyping methodologies, and statistical analyses. Sample sizes across the datasets ranged from 21,761 to 873,341, with all participants of European ancestry (Table 1).

**Table 1:** Summary information of the datasets.

Trait	Fullname	Author	Year	PMID	Consortium	Ncase	Ncontrol	N
Migraine	Migraine	Mitja I Kurki	2023	36653562	FinnGen	23,899	341,884	365,783
MA	Migraine with aura	Mitja I Kurki	2023	36653562	FinnGen	10,336	341,884	352,250
MO	Migraine without aura	Mitja I Kurki	2023	36653562	FinnGen	8626	341,884	350,510
Anxiety disorder	Anxiety disorder	Otowa	2016	26857599	PGC	7016	14,745	17,310
BD	Bipolar disorder	Mullins	2021	34002096	PGC	41,917	371,549	413,466
Epilepsy	Epilepsy	Abou-Khalil	2018	30531953	PGC	15,212	29,677	44,889
Insomnia	Insomnia	–	2018	–	Uk biobank	–	–	360,738
MDD	Major depressive disorder	Howard	2019	30718901	PGC	246,363	561,190	807,553
Stroke	Stroke	Malik	2018	29531354	PGC	40,585	406,111	446,696

BD = bipolar disorder, MA = migraine with aura, MDD = major depressive disorder, MO = migraine without aura, PGC = Psychiatric Genomics Consortium.

### 2.3. Selection of the IVs

In this study, we screened migraine and its subtypes as exposure phenotypes along with 6 neuropsychiatric disorders: anxiety, BD, epilepsy, MDD, insomnia, and stroke. To investigate the causal role of migraine in these disorders, we selected IVs that met the 3 core assumptions of MR analysis for robust results.

First, we selected SNPs significantly associated with migraine at genome-wide significance (*P* < 5 × 10^−8^). Given that only 5 SNPs met this threshold, which does not meet the basic requirements of MR operation, we relaxed it to *P* < 5 × 10^−6^ (Table S1, Supplemental Digital Content, https://links.lww.com/MD/Q705). Second, to avoid bias from linkage disequilibrium, we ensured SNP independence by setting thresholds where SNPs had pairwise linkage disequilibrium *R*^2^ < 0.001 and a physical distance > 10,000 kb.^[33]^ We also excluded palindromic SNPs with moderate allele frequencies.^[34]^ Third, to address weak IVs, we evaluated genetic instrument strength using the *F*-statistic for each SNP, calculated as [(N − *K* − 1)/*K*] × [*R*^2^/(1 − *R*^2^)], where N is the sample size, *K* is the number of IVs, and *R*^2^ is the proportion of variance explained. An *F*-statistic above 10 indicates a robust instrument validity. If *F* values were consistently below 10, we considered an alternative calculation: *F* = β^2^/σ^2^ (β: SNP exposure association beta, σ: variance).^[35]^ Ultimately, when migraine was the exposure factor, we identified SNPs associated with epilepsy, anxiety, BD, MDD, insomnia, and stroke (Table 2).

**Table 2:** Results of migraine and neuropsychiatric disorders screening SNPs.

Phenotypes		Outcome									
		Anxiety disorder	BD	Epilepsy	Insomnia	MDD	AS	AIS	LAS	CES	SVS
Exposure	Migraine	+	+	+	+	+	+	+	+	+	+
	MA	+	+	+	+	+	+	+	+	+	+
	MO	+	+	+	+	+	+	+	+	+	+

The “+” represents the selected relevant SNPs, while the “–” indicates that no relevant SNPs were selected.

AIS = any ischemic stroke, AS = any stoke, BD = bipolar disorder, CES = cardioembolic stroke, LAS = large artery stroke, MA = migraine with aura, MDD = major depressive disorder, MO = migraine without aura, SNPs = single nucleotide polymorphisms, SVS = small vessel stroke.

### 2.4. MR analysis

We conducted 2-sample MR analyses of migraine and 6 neuropsychiatric disorders using selected IVs with the 2-sample MR and MR-PRESSO packages in R package VISREG (v.4.4.0; R Foundation for Statistical Computing, Vienna, Austria). The primary method was the inverse variance weighting (IVW) method with random effects, while MR-Egger, weighted median, simple mode, and weighted mode served as auxiliary methods, resulting in 5 distinct analyses. The IVW method, assuming all IVs are valid and unaffected by horizontal pleiotropy, was used to estimate the causal relationship between exposure and outcome. Beta values were converted to OR with 95% confidence intervals (CI) for interpretability.

Sensitivity analyses were performed to assess heterogeneity (using Cochran *Q* and Rucker *Q* statistics, with *P* > .05 indicating no heterogeneity) and horizontal pleiotropy (using MR-Egger intercept test, with *P* > .05 suggesting no pleiotropy). MR-PRESSO was employed to identify outlier SNPs and further evaluate potential horizontal pleiotropy. Leave-one-out analysis was used to assess the influence of individual SNPs on the causal relationship, minimizing bias from pleiotropic effects. Publication bias was evaluated using funnel plot symmetry and directional pleiotropy assessments. Forest plots visualized the genetic effects, with combined effects estimated using MR-Egger regression and IVW. The Steiger test was used to analyze the direction of the deviation to refute the reverse causation. We used the Benjamini–Hochberg procedure to correct for multiple testing. A false discovery rate (FDR) adjusted *P*-value < .05 was regarded as statistically significant.

## 3. Results

### 3.1. Overview

A 2-sample MR analysis was conducted to investigate the causal associations between migraine and the risk of several neuropsychiatric disorders including anxiety, BD, epilepsy, MDD, insomnia, and stroke. And we used the IVW method, no significant associations were observed between migraine and the several neuropsychiatric disorders (all *P*_FDR_ > .05) (Table 3).

**Table 3:** Causal effects of migraine on the psychiatric disorders.

Exposure	Outcome	*b* (SE)	OR [95% CI]	N_IV	*P*val	*P*_FDR_
Migraine	Anxiety disorder	0.019 (0.015)	1.020 [0.990–1.050]	55	.199	.478
MA	Anxiety disorder	0.005 (0.018)	1.005 [0.970–1.041]	19	.785	1.000
MO	Anxiety disorder	0.007 (0.017)	1.007 [0.975–1.040]	18	.688	1.000
Migraine	BD	0.010 (0.046)	1.010 [0.923–1.105]	53	.835	1.000
MA	BD	0.121 (0.069)	1.129 [0.986–1.292]	17	.079	.296
MO	BD	0.073 (0.035)	1.075 [1.005–1.151]	18	.037	.185
Migraine	Epilepsy	-0.025 (0.020)	0.976 [0.938–1.014]	26	.215	.478
MA	Epilepsy	-0.029 (0.198)	0.972 [0.660–1.432]	7	.884	1.000
MO	Epilepsy	0.037 (0.033)	1.037 [0.973–1.106]	8	.266	.532
Migraine	MDD	0.035 (0.014)	1.036 [1.009–1.064]	61	.010	.077
MA	MDD	0.022 (0.019)	1.022 [0.985–1.060]	19	.243	.486
MO	MDD	0.013 (0.010)	1.013 [0.993–1.033]	20	.194	.478
Migraine	Insomnia	0.009 (0.005)	1.009 [0.999–1.019]	68	.088	.308
MA	Insomnia	0.007 (0.005)	1.007 [0.997–1.017]	24	.191	.478
MO	Insomnia	0.007 (0.004)	1.007 [0.999–1.016]	22	.099	.308
Migraine	AS	0.005 (0.025)	1.005 [0.957–1.056]	61	.832	1.000
MA	AS	0.033 (0.031)	1.033 [0.973–1.097]	23	.285	.532
MO	AS	-0.022 (0.029)	0.978 [0.925–1.035]	22	.445	.688
Migraine	AIS	0.012 (0.028)	1.013 [0.958–1.070]	61	.660	.990
MA	AIS	0.062 (0.035)	1.064 [0.993–1.141]	23	.078	.296
MO	AIS	-0.006 (0.030)	0.994 [0.937–1.055]	22	.851	1.000
Migraine	LAS	0.056 (0.072)	1.058 [0.919–1.258]	61	.434	.688
MA	LAS	0.108 (0.092)	1.115 [0.930–1.335]	23	.239	.532
MO	LAS	0.020 (0.069)	1.021 [0.891–1.691]	22	.768	1.000
Migraine	CES	0.051 (0.062)	1.052 [0.931–1.189]	61	.415	.688
MA	CES	0.068 (0.064)	1.070 [0.944–1.214]	23	.292	.688
MO	CES	-0.027 (0.070)	0.974 [0.850–1.116]	22	.700	1.000
Migraine	SVS	0.068 (0.073)	1.070 [0.928–1.235]	61	.350	.688
MA	SVS	0.025 (0.090)	1.025 [0.860–1.221]	23	.784	1.000
MO	SVS	-0.067 (0.060)	0.935 [0.831–1.052]	22	.263	.532

AIS = any ischemic stroke, AS = any stoke, BD = bipolar disorder, CES = cardioembolic stroke, CI = confidence intervals, IV = instrumental variable, LAS = large artery stroke, MA = migraine with aura, MDD = major depressive disorder, MO = migraine without aura, OR = odds ratios, SVS = small vessel stroke.

### 3.2. Causal relationship between migraine and anxiety disorder

In the MR analysis examining the causal relationship between migraine (including MA and MO) and anxiety disorder, 55 SNPs for migraine, 19 for MA, and 18 for MO were identified as IVs after screening and eliminating palindromic SNPs (Table S1, Supplemental Digital Content, https://links.lww.com/MD/Q705). The IVW method revealed no causal links between migraine, MA, or MO and anxiety disorder (migraine anxiety: OR = 1.020, 95% CI = [0.990–1.050], *P*_FDR_ = .478; MA–anxiety: OR = 1.005, 95% CI = [0.970–1.041], *P*_FDR_ = 1.000; MO–anxiety: OR = 1.007, 95% CI = [0.975–1.040], *P*_FDR_ = 1.000) (Table 3). Four additional methods also showed no significant causal relationships (*P*_FDR_ > .05). Thus, our MR analysis concluded no causal association between migraine and anxiety disorder. Scatter and forest plots of the MR analysis are presented in Figures S1b, S1b1, S1b2, and S4b, S4b1, S4b2, Supplemental Digital Content, https://links.lww.com/MD/Q705.

Heterogeneity results (*P* > .05) indicated no significant heterogeneity among the IVs (Table 4), and funnel plots for heterogeneity visualization are shown in Figures S2b, S2b1, and S2b2, Supplemental Digital Content, https://links.lww.com/MD/Q705. Horizontal pleiotropy was assessed using MR-Egger and MR-PRESSO, with no significant pleiotropy detected (*P* > .05) (Table 5). A leave-one-out analysis confirmed the stability of the results, as no single SNP significantly affected the robustness of the findings (Figures S3b, S3b1, and S3b2, Supplemental Digital Content, https://links.lww.com/MD/Q705). The Steiger test results showed that the effect of SNP on exposure factors was greater than that on outcome, and the direction test showed “true,” indicating that there was no reverse causal association (Table 6).

**Table 4:** The heterogeneity test of our MR analyses.

Exposure	Outcome	Heterogeneity test (MR-Egger)			Heterogeneity test (IVW)		
		Cochran *Q*	*Q*_df	*P*	Rucker *Q*	*Q*_df	*P*
Migraine	Anxiety disorder	62.068	53	.184	64.803	54	.149
MA	Anxiety disorder	9.008	17	.940	11.864	18	.854
MO	Anxiety disorder	17.942	16	.327	18.557	17	.355
Migraine	BD	71.421	51	.031	74.203	52	.023
MA	BD	30.452	15	.010	32.680	16	.008
MO	BD	15.928	16	.458	17.458	17	.424
Migraine	Epilepsy	4.540	24	.999	4.594	25	.999
MA	Epilepsy	0.164	5	.999	0.187	6	.999
MO	Epilepsy	8.346	6	.214	8.357	7	.302
Migraine	MDD	89.957	59	.006	92.475	60	.005
MA	MDD	30.039	17	.026	30.477	18	.033
MO	MDD	14.205	18	.716	14.227	19	.770
Migraine	Insomnia	104.662	66	.002	105.974	67	.002
MA	Insomnia	25.674	22	.266	26.134	23	.295
MO	Insomnia	26.992	20	.135	28.683	21	.122
Migraine	AS	59.640	59	.452	59.849	60	.481
MA	AS	23.663	21	.310	23.976	22	.348
MO	AS	33.123	20	.033	33.789	21	.038
Migraine	AIS	64.694	59	.285	65.203	60	.301
MA	AIS	26.594	21	.185	27.259	22	.202
MO	AIS	31.314	20	.051	32.317	21	.054
Migraine	LAS	67.104	59	.219	67.687	60	.231
MA	LAS	29.634	21	.099	29.648	22	.127
MO	LAS	26.026	20	.165	26.150	21	.201
Migraine	CES	82.039	59	.025	82.230	60	.030
MA	CES	23.565	21	.315	23.593	22	.369
MO	CES	42.121	20	.003	42.726	21	.003
Migraine	SVS	79.773	59	.037	80.070	60	.043
MA	SVS	30.212	21	.088	31.977	22	.078
MO	SVS	22.891	20	.294	23.041	21	.342

AIS = any ischemic stroke, AS = any stoke, BD = bipolar disorder, CES = cardioembolic stroke, LAS = large artery stroke, MA = migraine with aura, MDD = major depressive disorder, MO = migraine without aura, MR = Mendelian randomization, SVS = small vessel stroke.

**Table 5:** The horizontal pleiotropy test of our MR analyses.

Exposure	Outcome	Horizontal pleiotropy test (MR-Egger)		Horizontal pleiotropy test (MR-PRESSO)
		Intercept	*P*	Globle test *P*val
Migraine	Anxiety disorder	0.006	.132	.148
MA	Anxiety disorder	0.011	.109	.856
MO	Anxiety disorder	0.006	.470	.310
Migraine	BD	0.011	.165	.025
MA	BD	0.019	.311	.011
MO	BD	0.010	.234	.388
Migraine	Epilepsy	-0.002	.818	1.000
MA	Epilepsy	0.021	.885	.999
MO	Epilepsy	0.002	.934	.331
Migraine	MDD	0.003	.204	.002
MA	MDD	0.003	.625	.035
MO	MDD	-0.0004	.884	.796
Migraine	Insomnia	-0.0007	.366	.001
MA	Insomnia	-0.001	.537	.300
MO	Insomnia	0.111	.276	.153
Migraine	AS	0.002	.650	.492
MA	AS	0.005	.604	.344
MO	AS	-0.004	.533	.049
Migraine	AIS	0.003	.498	.301
MA	AIS	0.007	.477	.212
MO	AIS	-0.006	.433	.057
Migraine	LAS	-0.009	.477	.225
MA	LAS	-0.003	.924	.130
MO	LAS	0.005	.761	.212
Migraine	CES	0.004	.712	.026
MA	CES	0.003	.876	.375
MO	CES	-0.009	.598	.004
Migraine	SVS	-0.006	.641	.038
MA	SVS	0.028	.280	.083
MO	SVS	-0.005	.721	.341

AIS = any ischemic stroke, AS = any stoke, BD = bipolar disorder, CES = cardioembolic stroke, LAS = large artery stroke, MA = migraine with aura, MDD = major depressive disorder, MO = migraine without aura, SVS = small vessel stroke.

**Table 6:** The Steiger test of our MR analyses.

Exposure	Outcome	Steiger test	
		Causal direction	*P*val
Migraine	Anxiety disorder	TRUE	<.001
MA	Anxiety disorder	TRUE	<.001
MO	Anxiety disorder	TRUE	<.001
Migraine	BD	TRUE	<.001
MA	BD	TRUE	<.001
MO	BD	TRUE	<.001
Migraine	Epilepsy	TRUE	<.001
MA	Epilepsy	TRUE	<.001
MO	Epilepsy	TRUE	<.001
Migraine	MDD	TRUE	<.001
MA	MDD	TRUE	<.001
MO	MDD	TRUE	<.001
Migraine	Insomnia	TRUE	<.001
MA	Insomnia	TRUE	<.001
MO	Insomnia	TRUE	<.001
Migraine	AS	TRUE	<.001
MA	AS	TRUE	<.001
MO	AS	TRUE	<.001
Migraine	AIS	TRUE	<.001
MA	AIS	TRUE	<.001
MO	AIS	TRUE	<.001
Migraine	LAS	TRUE	<.001
MA	LAS	TRUE	<.001
MO	LAS	TRUE	<.001
Migraine	CES	TRUE	<.001
MA	CES	TRUE	<.001
MO	CES	TRUE	<.001
Migraine	SVS	TRUE	<.001
MA	SVS	TRUE	<.001
MO	SVS	TRUE	<.001

AIS = any ischemic stroke, AS = any stoke, BD = bipolar disorder, CES = cardioembolic stroke, LAS = large artery stroke, MA = migraine with aura, MDD = major depressive disorder, MO = migraine without aura, MR = Mendelian randomization, SVS = small vessel stroke.

### 3.3. The causal relationship between migraine and BD

In the MR analysis examining the causal relationship between migraine (including MA and MO) and BD, 53, 17, and 18 genome-wide significant IVs were selected for migraine, MA, and MO, respectively, after filtering and excluding unmatched abnormal SNPs (Table S1, Supplemental Digital Content, https://links.lww.com/MD/Q705). The IVW method showed no causal relationship between migraine, MA or MO and BD (migraine–BD: OR = 1.010, 95% CI = [0.923–1.105], *P*_FDR_ = 1.000; MA–BD: OR = 1.129, 95% CI = [0.986–1.292], *P*_FDR_ = .296; MO–BD: OR = 1.075, 95% CI = [1.005–1.151], *P*_FDR_ = .185) (Table 3). Scatter and forest plots of the MR analysis results are presented in Figures S1c, S1c1, S1c2, and S4c, S4c1, S4c2, Supplemental Digital Content, https://links.lww.com/MD/Q705.

For MO versus BD, no heterogeneity was detected among the included IVs (*P* > .05) (Table 4). Horizontal pleiotropy was assessed using MR-Egger and MR-PRESSO, with no significant pleiotropy detected (*P* for MR-Egger intercept > .05) (Table 5). Detailed visual analyses are provided in the leave-one-out plots (Figures S3c, S3c1, and S3c2, Supplemental Digital Content, https://links.lww.com/MD/Q705). The Steiger test results show that the direction is “true,” indicating that there is no reverse causal association (Table 6).

### 3.4. Causal relationship between migraine and epilepsy

In the MR analysis of the causal relationship between migraine (including MA and MO) and epilepsy, 26, 7, and 8 genome-wide significant IVs were identified for migraine, MA, and MO, respectively (Table S1, Supplemental Digital Content, https://links.lww.com/MD/Q705). The IVW method indicated no causal association between migraine or its subtypes and epilepsy (migraine–epilepsy: OR = 0.976, 95% CI = [0.938–1.014], *P*_FDR_ = .478 MA–epilepsy: OR = 0.972, 95% CI = [0.660–1.432], *P*_FDR_ = 1.000; MO–epilepsy: OR = 1.037, 95% CI = [0.973–1.106], *P*_FDR_ = .532) (Table 3). Consistent results from four additional methods supported the absence of a causal relationship (*P*_FDR_ > .05). Thus, the MR analysis concluded no causal link between migraine and epilepsy. Scatter and forest plots of the MR analysis results are presented in Figures S1a, S1a1, S1a2, and S4a, S4a1, S4a2, Supplemental Digital Content, https://links.lww.com/MD/Q705.

For migraine, MA, and MO versus epilepsy, Cochran *Q* and Rucker *Q P* > .05 indicated no heterogeneity (Table 4). Funnel plots for heterogeneity visualization are shown in Figures S2a, S2a1, and S2a2, Supplemental Digital Content, https://links.lww.com/MD/Q705. Horizontal pleiotropy was assessed using MR-Egger and MR-PRESSO, with no significant pleiotropy detected (*P* > .05) (Table 5). Detailed visual analyses are provided in the leave-one-out plots (Figures S3a, S3a1, S3a2, Supplemental Digital Content, https://links.lww.com/MD/Q705). The Steiger test results show that the direction of exposure to the outcome is “true” and there is no reverse causal association (Table 6).

### 3.5. Causal relationship between migraine and MDD

In the MR analysis examining the causal relationship between migraines (including MA and MO) and MDD, we selected 61, 19, and 20 genome-wide significant IVs for migraine, MA, and MO, respectively, after screening and eliminating abnormal IVs (Table S1, Supplemental Digital Content, https://links.lww.com/MD/Q705). The IVW method also indicated no causal association between migraine or its subtypes and MDD (migraine–MDD: OR = 1.036, 95% CI = [1.009–1.064], *P*_FDR_ = .077; MA–MDD: OR = 1.022, 95% CI = [0.985–1.060], *P*_FDR_ = .486; MO–MDD: OR = 1.013, 95% CI = [0.993–1.033], *P*_FDR_ = .478) (Table 3). Additionally, no causal association was found using the other 4 methods (*P*_FDR_ > .05). Scatter and forest plots of the MR analysis results are presented in Figures S1d, S1d1, and S1d2, and S4d, S4d1, and S4d2, Supplemental Digital Content, https://links.lww.com/MD/Q705.

For MO versus MDD, no heterogeneity was detected (*P* > .05), suggesting no need to consider heterogeneity’s impact (Table 4). Funnel plots for heterogeneity visualization are shown in Figures S2d, S2d1, and S2d2, Supplemental Digital Content, https://links.lww.com/MD/Q705. Horizontal pleiotropy was assessed using MR-Egger and MR-PRESSO, with no significant pleiotropy detected (*P* for MR-Egger intercept > .05) (Table 5). Detailed visual analyses are provided in the leave-one-out plots (Figures S3d, S3d1, and S3d2, Supplemental Digital Content, https://links.lww.com/MD/Q705). The Steiger test results show that the direction of exposure to the outcome is “true” and there is no reverse causal association (Table 6).

### 3.6. Causal relationship between migraine and insomnia

In the causal analysis between migraine and insomnia, 68, 24, and 22 genome-wide significant IVs were selected for migraine, MA, and MO, respectively, after removing abnormal IVs (Table S1, Supplemental Digital Content, https://links.lww.com/MD/Q705). The IVW method showed no causal association between migraine or its subtypes and insomnia (migraine–insomnia: OR = 1.009, 95% CI = [0.999–1.019], *P*_FDR_ = .308; MA–insomnia: OR = 1.007, 95% CI = [0.997–1.017], *P*_FDR_ = .478; MO–insomnia: OR = 1.007, 95% CI = [0.999–1.016], *P*_FDR_ = .308) (Table 3). Consistent results from 4 additional methods supported the absence of a causal relationship (*P*_FDR_ > .05). Scatter and forest plots of the MR analysis results are presented in Figures S1j, S1j1, and S1j2 and S4j, S4j1, and S4j2, Supplemental Digital Content, https://links.lww.com/MD/Q705.

No heterogeneity was detected for MA and MO versus insomnia (Table 4). Funnel plots for heterogeneity visualization are shown in Figures S2j, S2j1, and S2j2, Supplemental Digital Content, https://links.lww.com/MD/Q705. Horizontal pleiotropy was assessed using MR-Egger and MR-PRESSO, with no significant pleiotropy detected (*P* > .05) (Table 5). Detailed visual analyses are provided in the leave-one-out plots (Figures S3j, S3j1, and S3j2, Supplemental Digital Content, https://links.lww.com/MD/Q705). The Steiger test results show that the direction of exposure to the outcome is “true” and there is no reverse causal association (Table 6).

### 3.7. Causal effects of migraine on stroke and its subtypes

In the MR analysis examining the causal relationships between migraine (including MA and MO) and any stroke (AS) and its subtypes (any ischemic stroke [AIS], LAS, CES, SVS), a total of 61, 23, and 22 genome-wide significant associations IVs were selected for migraine, MA, and MO, respectively (Table S1, Supplemental Digital Content, https://links.lww.com/MD/Q705). The IVW method indicated no causal association between migraine (including both MA and MO) and stroke or its subtypes (migraine–AS: OR = 1.005, 95% CI = [0.957–1.056], *P*_FDR_ = 1.000; MA–AS: OR = 1.033, 95% CI = [0.973–1.097], *P*_FDR_ = .532; MO–AS: OR = 0.978, 95% CI = [0.925–1.035], *P*_FDR_ = .688). Migraine–AIS: OR = 1.013, 95% CI = [0.958–1.070], *P*_FDR_ = .990; MA–AIS: OR = 1.064, 95% CI = [0.993, 1.141], *P*_FDR_ = .296; MO–AIS: OR = 0.994, 95% CI = [0.937–1.055], *P*_FDR_ = 1.000. Migraine–LAS: OR = 1.058, 95% CI = [0.919–1.258], *P*_FDR_ = .688; MA–LAS: OR = 1.115, 95% CI = [0.930–1.335, *P*_FDR_ = .532; MO–LAS: OR = 1.021 95% CI = [0.891–1.691], *P*_FDR_ = 1.000. Migraine–CES: OR = 1.052, 95% CI = [0.931–1.189], *P*_FDR_ = .688; MA–CES: OR = 1.070, 95% CI = [0.944–1.214], *P*_FDR_ = .688; MO–CES: OR = 0.974, 95% CI = [0.850–1.16], *P*_FDR_ = 1.000. Migraine–SVS: OR = 1.070, 95% CI = [0.928–1.235], *P*_FDR_ = .688; MA–SVS: OR = 1.025, 95% CI = [0.860–1.221], *P*_FDR_ = 1.000; MO–SVS: OR = .935, 95% CI = [0.831–1.052], *P*_FDR_ = .532) (Table 3). The other 4 methods also demonstrated no significant results (*P*_FDR_ > .05). Thus, the MR analysis supported the assertion that no causal link exists between migraine and stroke, including its various subtypes. Scatter plots of the MR analysis results are presented in Figures S1e, S1e1, S1e2, Supplemental Digital Content, https://links.lww.com/MD/Q705; S1f, S1f1, S1f2, Supplemental Digital Content, https://links.lww.com/MD/Q705; S1g, S1g1, S1g2, Supplemental Digital Content, https://links.lww.com/MD/Q705; S1h, S1h1, S1h2, Supplemental Digital Content, https://links.lww.com/MD/Q705; S1i, S1i1, S1i2, Supplemental Digital Content, https://links.lww.com/MD/Q705. And forest plots of the MR analysis results are presented in and Figures S4e, S4e1, S4e2, Supplemental Digital Content, https://links.lww.com/MD/Q705; S4f, S4f1, S4f2, Supplemental Digital Content, https://links.lww.com/MD/Q705; S4g, S4g1, S4g2, Supplemental Digital Content, https://links.lww.com/MD/Q705; S4h, S4h1, S4h2, Supplemental Digital Content, https://links.lww.com/MD/Q705; S4i, S4i1, S4i2, Supplemental Digital Content, https://links.lww.com/MD/Q705.

For migraine versus stroke and its subtypes, except for MO–AS, migraine–CES, MO–CES, and migraine–SVS, no significant heterogeneity was observed (Table 4). Funnel plots illustrating heterogeneity are shown in Figures S2e, S2e1, S2e2, Supplemental Digital Content, https://links.lww.com/MD/Q705; Figures S2f, S2f1, S2f2, Supplemental Digital Content, https://links.lww.com/MD/Q705; Figures S2g, S2g1, S2g2, Supplemental Digital Content, https://links.lww.com/MD/Q705; Figures S2h, S2h1, S2h2, Supplemental Digital Content, https://links.lww.com/MD/Q705; Figures S2i, S2i1, S2i2, Supplemental Digital Content, https://links.lww.com/MD/Q705. Horizontal pleiotropy was assessed using MR-Egger and MR-PRESSO, with no significant pleiotropy detected (Table 5). Detailed visualization are provided in the leave-one-out plots (Figures S3e, S3e1, S3e2, Supplemental Digital Content, https://links.lww.com/MD/Q705; Figures S3f, S3f1, S3f2, Supplemental Digital Content, https://links.lww.com/MD/Q705; Figures S3g, S3g1, S3g2, Supplemental Digital Content, https://links.lww.com/MD/Q705; Figures 3h, S3h1, S3h2, Supplemental Digital Content, https://links.lww.com/MD/Q705; Figures S3i, S3i1, S3i2, Supplemental Digital Content, https://links.lww.com/MD/Q705). The Steiger test results indicated that the direction of causality was from exposure to outcome, with no evidence of reverse causation (Table 6).

## 4. Discussion

Our study investigated the causal relationships between migraine and several neuropsychiatric disorders using MR. We did not find any significant causal associations between migraine and the several neuropsychiatric disorders.

Although epidemiological and clinical studies have demonstrated a high degree of comorbidity between BD and migraine, suggesting that both conditions may share multifactorial and polygenic etiologies, as well as common pathophysiological mechanisms.^[36]^ We found no association between genetically predicted migraine and BD risk. Our findings suggest that migraine and BD are not primary causative factors for each other, while traditional epidemiological studies may have produced false results due to residual confounding factors. For example, their underlying overlapping neurobiological mechanisms may interpret the association of comorbidities found in epidemiology. Shared pathophysiological mechanisms, such as glutamate dysregulation and inflammation, may underlie this relationship.^[37,38]^

Study has demonstrated that migraine patients with aura exhibit increased platelet glutamate uptake, whereas those without aura display decreased platelet glutamate uptake. This suggests that glutamate may contribute to the pathogenesis of migraine by modulating the balance of neurotransmitters. Moreover, significant alterations in brain glutamate levels have been observed in patients with BD. For instance, research has shown that during the remission phase, patients with BD exhibit a significant upregulation of glutamate levels in the anterior cingulate cortex, while a downregulation is observed in the hippocampal region.^[39]^ These aberrations in glutamate levels may be associated with neuronal excitability and synaptic plasticity, thereby influencing emotional regulation and cognitive function.^[40]^ On the other hand, inflammatory mediators, such as cytokines and chemokines, can activate the trigeminovascular system, leading to vasodilation and neuroinflammation, thereby triggering migraine attacks.^[41]^ Moreover, inflammation may further exacerbate migraine symptoms by influencing the metabolism and release of neurotransmitters.^[42]^ Similarly, inflammation plays a pivotal role in the pathogenesis of BD. Studies have revealed that patients with BD exhibit elevated levels of inflammatory markers, such as interleukin-6 and C-reactive protein. The increase in these inflammatory markers may be associated with neuroinflammation and alterations in synaptic plasticity, which in turn can affect emotional regulation and cognitive function.^[39,42]^ Therefore, the aforementioned pathological mechanisms may lead to cross-sensitization between BD and migraine, this also explains why our genetic findings do not confirm that migraine is a risk factor for BD.

Although we did not reveal that migraine is a risk factor for MDD under the strictest Benjamini–Hochberg–FDR criteria, we found consistent signals for the association between migraine and MDD at relatively lax thresholds (e.g., original *P* < .05). Migraine and MDD are both polygenic disorders, and a wealth of data indicates a shared genetic connection.^[43,44]^ Research has demonstrated that genetics plays a significant role in the common comorbidity between migraine and MDD,^[45]^ providing a robust foundational starting point for investigating the intricately intertwined mechanisms at various analytical levels.^[44]^ Building on this research, scholars have utilized SNP and GWAS gene data analyses to uncover a significant genetic overlap between these 2 disorders, revealing the commonality of genetic risk factors for both conditions.^[46]^ Furthermore, by evaluating the expression of overlapping genes, it has been discovered that 4 cell types exhibit a shared gene expression pattern between the 2 disorders, including microglia, astrocytes, and neurons within the central nervous system, as well as peptidergic nociceptors expressing CGRP and substance P in the trigeminal ganglion. This study further identified 8 functional categories of overlapping gene expression between the 2 disorders, including the synthesis/binding of neuropeptides, dopaminergic and serotonergic neurotransmitters, glutamatergic neurotransmitters and endocannabinoid genes, calcium channels, inflammatory factors and immune responses, hormone genes, vascular regulation, and drug metabolism.^[47]^ This further suggests that the comorbidity of migraine and MDD may be caused by their shared neurological symptoms. On this basis, our results highlight the importance of vigilant assessment and management of MDD in migraine patients and suggest that future research should focus on dissecting the molecular mechanisms within different migraine subtypes.

The findings of this study do not support a direct causal link between migraine and epilepsy, seemingly contradicting previous research that identified shared genetic mutations in genes such as CACNA1A, ATP1A2, and SCN1A implicated in both conditions.^[48]^ This genetic overlap suggests a predisposition for coexistence rather than causal interaction.^[49]^ For instance, a large-scale GWAS identified 3 susceptibility genes for MO, with LRP1 being a lipoprotein associated with glutamate receptors.^[50]^ Although glutamate has excitatory properties, it appears unrelated to epilepsy. Other research has found that adult epilepsy patients exhibit lower comorbidity rates with migraines but higher co-morbidity rates with anxiety, depression, and sleep disorders, with these 3 conditions serving as mutual risk factors.^[51]^

The absence of a direct causal link implies that observed comorbidities may stem from shared environmental influences or lifestyle behaviors rather than a direct effect of 1 condition on the other.^[52]^ Epileptic seizures are typically triggered by multiple factors. Generalized seizures are associated with sleep disturbances, emotional fluctuations, stress, and irregular lifestyles, while focal seizures are linked to stress, mood swings, sleep irregularities, exposure to cold, and overeating. Analysis of these triggers reveals that sleep deprivation is closely related to anger, fatigue, and high stress levels.^[53]^ For instance, ischemic events are known to increase the risk of developing either migraine or epilepsy.^[13]^ Additionally, common triggers, such as stress, sleep deprivation, and fatigue, are frequently reported in patients with idiopathic/genetic epilepsy and migraines, indicating potential shared pathophysiological mechanisms.^[54]^ These findings underscore the complexity of the relationship between migraine and epilepsy and suggest that a multifactorial approach is necessary to fully understand their comorbidities. The therapeutic implications are significant: targeted therapies effective for 1 condition may not exert the same influence on the other. Therefore, future research should concentrate on elucidating the underlying genetic and neurochemical factors that could account for the observed comorbidity without implying a direct causal relationship. Such investigations are crucial for developing tailored treatment strategies to address the unique pathophysiological features of each condition.

No significant causal associations were found between migraine and stroke in our study’s results, including subtypes such as ischemic, large artery, cardioembolic, and small-vessel stroke. The absence of a direct causal relationship is consistent with previous research, which indicates that the association between migraine and stroke may be confounded by vascular risk factors.^[55]^ The implications of our findings are unequivocal: migraine-specific pharmacological interventions are not requisite for the primary prevention of stroke in individuals with migraines. Furthermore, migraine has not yet been identified as a modifiable risk factor for stroke.^[56]^ Our results also suggested that any associations observed in epidemiological studies might be attributed to residual confounders, such as shared vascular risk factors, rather than a direct causal link. For example, migraine patients often carry a higher burden of traditional vascular risk factors (such as high blood pressure, hyperlipidemia, and smoking). These factors may play an important role in the association between migraines and strokes.^[57]^ This insight is pivotal for the development of targeted prevention strategies for stroke, indicating that interventions may be more effectively focused on vascular risk factors than treating migraine as a primary means to prevent stroke.

The present study did not identify a significant causal association between migraine and insomnia, in addition to the complexity of this relationship. Our findings are consistent with those of previous research that reported a high prevalence of migraine among subjects with insomnia.^[58,59]^ The lack of a direct causal link implies that the observed association may stem from shared mechanisms or risk factors such as emotional stress or depression,^[60]^ rather than a direct causal effect. Migraines and sleep disorders are likely to share physiological mechanisms such as structural and neurotransmitter dysregulation in the central nervous system.^[61]^ This highlights the need for a holistic treatment approach that addresses both sleep hygiene and migraine management in patients with comorbid insomnia and migraine.

Our study has several limitations. First, there may be some overlap among participants in the GWAS data, potentially introducing bias. However, the *F*-test values exceeded 10, suggesting minimal sample overlap. Second, the GWAS data were derived exclusively from European populations, limiting the generalizability of our findings. Future research should validate these results in diverse populations. Third, our analysis did not include gender stratification, precluding the examination of sex-based differences. Future GWAS databases should include gender-specific information. Lastly, mild cases of migraine or neuropsychiatric disorders may be underrepresented in our data, potentially affecting the robustness of our findings.

## 5. Conclusion

Although our study does not provide insights into the causal relationships between migraine and various neuropsychiatric disorders. However, we emphasize the importance of exploring the shared pathophysiological mechanisms migraine and neuropsychiatric disorders. Future research should focus on elucidating these mechanisms, validating our findings in diverse populations, and developing targeted treatment strategies to address the unique pathophysiological features of each condition.

## Acknowledgments

We would like to express our sincere gratitude to the Psychiatric Genomics Consortium (PGC) and the FinnGen project for providing publicly available GWAS summarized data for neuropsychiatric disorders and migraine, respectively. Additionally, we extend our appreciation to the MEGASTROKE project for providing publicly available GWAS summarized data for stroke and its subtypes.

## Author contributions

**Data curation:** Bing Deng.

**Methodology:** Qinlong Gao, Qing Ren.

**Software:** Genfa Du.

**Writing – original draft:** Tiantian Xu.

**Writing – review & editing:** Lingsan Hu, Dan Xi.

## Correction

The first two affiliations, 'a' and 'b,’ have been swapped. The current affiliations have been updated online as follows; *^a^Shenzhen Bao'an Traditional Chinese Medicine Hospital, Guangzhou University of Chinese Medicine, Shenzhen, Guangdong, China, ^b^Guangzhou University of Chinese Medicine, Guangzhou, Guangdong, China.* Also the affiliation links in the Author group have been updated accordingly to reflect this change.

## Supplementary Material


