# Supplementary material for: Causal influences of migraine on neuropsychiatric disorders: A 2-sample Mendelian randomization study
Source: Medicine (Baltimore). 2025 Dec 19;104(51):e46041. doi: 10.1097/MD.0000000000046041 (PMC12727394; doi:10.1097/MD.0000000000046041)
Supplement: Supplementary file 1 [file medi-104-e46041-s001.pdf]

Supplementary Table 1:

Instrumental Variable

1. Migraine-Anxiety disorder

SNP	EA	OA	Beta	SE	Pval
rs1038082	C	T	0.153775803	0.228640339	0.501223202
rs10768192	T	C	0.091141086	0.097499767	0.349900344
rs10864152	T	C	0.102678845	0.121069086	0.396381577
rs10909892	A	G	0.204189978	0.288341	0.478849314
rs11129828	T	C	0.187490412	0.098479813	0.056930224
rs111503046	C	T	-0.047963356	0.083935873	0.567709166
rs11172048	T	C	-0.040558721	0.118708452	0.73260176
rs11200607	T	C	-0.084862717	0.072341988	0.24076496
rs12779494	T	C	0.023036697	0.090501311	0.799074193
rs12948758	A	G	0.154830878	0.185797054	0.404656762
rs1379828	T	C	0.032882782	0.096917672	0.734394503
rs1557343	T	A	0.255411651	0.089487294	0.004314989
rs2001425	T	C	0.266817594	0.151600906	0.078407807
rs2112563	G	A	-0.059939837	0.089212781	0.501663277
rs2183947	A	G	-0.20843634	0.087252421	0.016899412
rs2462601	C	T	-0.01109974	0.1109974	0.920344325
rs2541318	A	G	0.118597193	0.126833109	0.349754836
rs2589253	T	C	-0.134445457	0.090219978	0.136172687

rs28448367	T	C	0.035520119	0.133722801	0.79052803
rs2976950	A	G	-0.176899604	0.090372624	0.05029496
rs2981889	C	T	0.013826751	0.120490257	0.908640033
rs35231114	A	G	-0.029288642	0.117154567	0.802587349
rs35340064	C	A	-0.005169101	0.196425825	0.979005461
rs36010570	C	G	0.037520892	0.072995191	0.607238922
rs37634	T	C	0.130622783	0.142148322	0.358137984
rs4661616	A	T	0.054716346	0.101326567	0.589197032
rs4683465	C	T	0.15407315	0.085193389	0.070527063
rs4888422	G	A	-0.061594629	0.08585918	0.473132684
rs4910165	G	C	0.075627493	0.08250272	0.359317338
rs529623	C	T	0.12965883	0.112928658	0.25090741
rs582161	A	G	0.218086977	0.111268866	0.04999579
rs6112036	A	G	0	0.101658807	1
rs62435157	A	G	-0.052245717	0.159349436	0.743010825
rs6713608	T	G	0.008627011	0.097053876	0.929170214
rs6891352	C	T	-0.118805909	0.08529655	0.163662994
rs71330635	G	T	-0.033885311	0.104832681	0.746519303
rs72704264	C	G	-0.062632001	0.090306605	0.487965471
rs72745900	A	G	0.121044384	0.088329686	0.170571316
rs72829857	G	A	0.082929818	0.079858344	0.299055233
rs7292782	C	A	0.033980203	0.083251498	0.683153815

rs7367621	G	A	0.088161545	0.096362619	0.360247481
rs73762250	G	C	-0.238102601	0.145335354	0.101359571
rs7398375	G	C	0.12984758	0.105689891	0.219232533
rs74522625	G	A	0.014773298	0.32842178	0.964121099
rs7502966	T	C	0.025467974	0.125217539	0.838830348
rs7829017	C	T	0.043082634	0.086165269	0.617075077
rs7957385	G	A	-0.133072301	0.101495823	0.189820227
rs869408	T	A	-0.05262213	0.094410292	0.577269843
rs923688	T	C	0.110435134	0.147904198	0.455264795
rs9266329	A	G	-0.907189651	0.61114112	0.137697727
rs9305545	G	A	0.126037038	0.109005006	0.247578914
rs9349379	G	A	-0.019259065	0.064196884	0.764177156
rs9423933	G	A	-0.143885755	0.155876234	0.35596712
rs9486715	C	A	0.184990458	0.091521595	0.043251141
rs9653353	T	C	-0.003624311	0.081547001	0.964550135

2. MA-Anxiety disorder

SNP	EA	OA	Beta	SE	Pval
rs10864152	T	C	0.069093605	0.081468579	0.396381577
rs11232520	G	T	0.031608196	0.067526601	0.63972373
rs12568138	A	C	0.015247695	0.075435964	0.839816925
rs12950737	A	G	0.075531652	0.061206683	0.217187048

rs13389386	C	T	0.027261601	0.068871413	0.692227985
rs17564412	A	G	-0.035949104	0.096494962	0.709484122
rs35356792	G	A	-0.091967146	0.081660483	0.260075122
rs35695296	A	T	0.042460496	0.069480811	0.541126024
rs4721922	G	A	0.071139914	0.095235691	0.455070834
rs653429	A	G	-0.066247483	0.162738383	0.683949492
rs6773261	G	A	0.029786561	0.067950592	0.661128118
rs6850727	C	G	-0.068815547	0.0587226	0.241247246
rs7096340	C	G	-0.037072589	0.065539042	0.571627267
rs74466079	A	G	-0.069296545	0.112527417	0.538013848
rs755869	G	A	-0.132445206	0.126492613	0.295072449
rs8180401	A	G	-0.043848687	0.094274677	0.641848189
rs921038	C	T	0.106723907	0.07064822	0.130880631
rs9261647	A	G	-0.247344289	0.272186729	0.363492581
rs9349379	G	A	-0.019902293	0.066340977	0.764177156

3. MO-Anxiety disorder

SNP	EA	OA	Beta	SE	Pval
rs10774231	C	T	0.008697759	0.084492517	0.918009657
rs11072103	G	T	0.107838069	0.069157023	0.118920179
rs12246748	T	C	0.02058272	0.061748159	0.73888268
rs1458265	A	T	-0.082000603	0.087820001	0.350440637

rs1509564	T	G	-0.04592925	0.064841294	0.478738282
rs2318311	G	A	-0.009159546	0.074584876	0.902259917
rs2403304	C	T	-0.060858636	0.063504664	0.3378947
rs2500283	C	G	0.066341069	0.081083529	0.413253375
rs55914748	G	T	-0.081418936	0.120724629	0.500045218
rs59996663	A	G	-0.123083888	0.055175536	0.025696419
rs61903808	C	A	-0.097836521	0.176290334	0.578912566
rs67422184	T	C	-0.029163245	0.072908113	0.689156517
rs72829868	A	G	0.104611265	0.085362792	0.220390582
rs7398375	G	C	0.090529842	0.07368708	0.219232533
rs77276355	A	G	0.002970062	0.068311421	0.965320293
rs9349379	G	A	-0.015499759	0.051665862	0.764177156
rs940659	C	T	0.018669182	0.055024958	0.734394503
rs9486715	C	A	0.0849367	0.042021315	0.043251141

4. Migraine-Bipolar disorder

SNP	EA	OA	Beta	SE	Pval
rs1038082	C	T	0.317721927	0.430976921	0.460992634
rs10768192	T	C	0.052922878	0.28826018	0.854331867
rs10864152	T	C	-0.173230647	0.243670692	0.477133103
rs10870513	T	C	0.646511775	0.463677626	0.163223086
rs10909892	A	G	-0.0569327	0.33289154	0.864204286

rs11129828	T	C	-0.089030155	0.28218254	0.752377845
rs111503046	C	T	0.130704635	0.242214948	0.589457398
rs11172048	T	C	-0.136562698	0.329415954	0.678463992
rs11200607	T	C	-0.075060635	0.219808349	0.732740614
rs117715021	T	C	0.169250006	0.48026712	0.724532256
rs118113255	T	C	0.213425021	0.238385094	0.370629327
rs12202086	C	A	-0.054400931	0.329455305	0.868846495
rs12779494	T	C	0.404789607	0.25504915	0.112489021
rs12811859	T	G	-0.325565171	0.297239915	0.273387743
rs12948758	A	G	0.143940878	0.326693153	0.659502881
rs1379828	T	C	0.31153928	0.289022343	0.281075149
rs145880920	A	C	-0.145661263	0.341063271	0.669321142
rs147855385	T	C	0.147483958	0.321547681	0.646471869
rs186941956	A	G	-0.10764006	0.205852081	0.601043821
rs2001425	T	C	0.264702687	0.278945666	0.342651154
rs2112563	G	A	0.567295742	0.256486745	0.026980929
rs2183947	A	G	-0.452487767	0.25206255	0.07263127
rs2462601	C	T	-0.510644463	0.304132877	0.093148607
rs2541318	A	G	-0.092170219	0.360733128	0.798330725
rs2589253	T	C	-0.230046598	0.270659933	0.395354552
rs28448367	T	C	-0.198592714	0.357290609	0.578327314
rs2976950	A	G	0.315433222	0.263426584	0.23114147

rs2981889	C	T	0.545084325	0.318015269	0.086525172
rs35231114	A	G	-0.250998187	0.297070508	0.398160445
rs35340064	C	A	0.326910666	0.443250382	0.460799816
rs37634	T	C	-0.180645163	0.291980337	0.536120943
rs4683465	C	T	-0.344369486	0.255580167	0.177850472
rs4888422	G	A	0.347095389	0.251978028	0.168363041
rs529623	C	T	0.232160264	0.28859546	0.421137843
rs582161	A	G	0.251377725	0.329355842	0.445319966
rs6112036	A	G	0.024323095	0.30497642	0.936432896
rs62435157	A	G	-1.054109941	0.365720018	0.003948003
rs6891352	C	T	0.131035132	0.261982261	0.616956821
rs71330635	G	T	-0.86835197	0.296496471	0.003403726
rs72745900	A	G	0.346828272	0.260081852	0.182356274
rs72829857	G	A	-0.25800908	0.242646506	0.287640225
rs7292782	C	A	-0.185198469	0.244657463	0.449067567
rs7367621	G	A	0.145468822	0.282937051	0.607155183
rs74522625	G	A	-0.948913723	0.409106715	0.020369173
rs7502966	T	C	0.093389033	0.301371026	0.75665176
rs76710367	T	C	-0.111410298	0.141217801	0.430155662
rs7829017	C	T	0.201097432	0.253110476	0.426901747
rs7957385	G	A	0.315799057	0.304487469	0.299666141
rs923688	T	C	-0.146903031	0.297780451	0.621781805

rs9305545	G	A	0.720497506	0.320202204	0.024440512
rs9423933	G	A	0.689483369	0.289270319	0.017147666
rs9486715	C	A	-0.122680075	0.276512054	0.657281262
rs9653353	T	C	-0.065301019	0.242828847	0.787992878

5. MA-Bipolar disorder

SNP	EA	OA	Beta	SE	Pval
rs10864152	T	C	-0.116568607	0.163968407	0.477133103
rs11232520	G	T	0.337691469	0.201143067	0.093178925
rs12568138	A	C	0.255200768	0.195009991	0.190651296
rs12950737	A	G	0.243574356	0.17971324	0.175306044
rs13389386	C	T	0.714536224	0.199440133	0.000340048
rs142786837	C	T	0.329326552	0.233992937	0.159302615
rs151255285	A	C	0.118269582	0.195635455	0.545484261
rs17564412	A	G	-0.376495623	0.183529634	0.040226455
rs35356792	G	A	-0.187874569	0.233881965	0.421808308
rs4721922	G	A	0.088354357	0.188176546	0.638691516
rs4998284	G	A	-0.014702674	0.18310245	0.936000626
rs653429	A	G	0.610605234	0.216024402	0.00470514
rs6773261	G	A	0.304370498	0.204782605	0.137197059
rs74466079	A	G	0.027332264	0.220604596	0.90139677
rs755869	G	A	-0.267823207	0.241080039	0.266598197

rs8180401	A	G	0.117322897	0.185260702	0.526547291
rs921038	C	T	-0.01955371	0.21194466	0.92649261

6. MO-Bipolar disorder

SNP	EA	OA	Beta	SE	Pval
rs10774231	C	T	0.141707846	0.168985034	0.401703789
rs11072103	G	T	0.205080435	0.19223308	0.286047628
rs114974197	A	G	0.016550048	0.21602568	0.938932624
rs116906873	C	T	0.279050723	0.20113394	0.165323622
rs117365086	A	G	0.412557318	0.206010539	0.04521992
rs12246748	T	C	0.254959692	0.171522664	0.137160397
rs1509564	T	G	0.233692037	0.189120441	0.216578143
rs151099105	C	T	0.210129933	0.125843346	0.094964109
rs2318311	G	A	0.252574516	0.213286576	0.236332876
rs3176321	T	C	-0.026348486	0.063916988	0.680171015
rs55914748	G	T	-0.074425209	0.203547339	0.714633137
rs59996663	A	G	0.275194035	0.153501171	0.073007894
rs61903808	C	A	0.091397896	0.149523739	0.5410274
rs72829868	A	G	-0.164037557	0.232655452	0.480768876
rs77276355	A	G	0.128655111	0.192063995	0.502949949
rs78872282	C	A	0.02796375	0.114245257	0.806634944
rs940659	C	T	-0.182722333	0.170970404	0.285188363

rs9486715	C	A	-0.056327449	0.126958014	0.657281262

7. Migraine-Epilepsy

SNP	EA	OA	Beta	SE	Pval
rs10864152	T	C	0.102649516	0.882731564	0.907425707
rs10870513	T	C	-0.298585767	1.306073313	0.819169464
rs11129828	T	C	0.259945959	0.424220731	0.540034319
rs11200607	T	C	-0.039707617	0.146075169	0.785752719
rs12779494	T	C	0.012416452	0.421242468	0.976485132
rs1379828	T	C	-0.173304	0.979560755	0.859571139
rs1557343	T	A	-0.168733283	0.480994204	0.725737592
rs2112563	G	A	-0.160119063	0.100364378	0.110627862
rs2183947	A	G	-0.033139167	0.279530905	0.905629675
rs2541318	A	G	0.074723732	1.095376851	0.945612597
rs2589253	T	C	-0.110720926	0.689917475	0.872499584
rs36010570	C	G	-0.021881248	0.021148139	0.300825194
rs4683465	C	T	-0.172150106	0.210264535	0.412939869
rs4910165	G	C	0.119028831	1.19628944	0.920742595
rs6112036	A	G	0.037621586	0.656362294	0.954291625
rs6713608	T	G	0.061778393	0.4248803	0.884393583
rs6891352	C	T	-0.034011838	1.579509332	0.982820346
rs72704264	C	G	0.037181489	0.254897677	0.884025362

rs7292782	C	A	0.059612436	0.98032886	0.951511633
rs7367621	G	A	0.134172126	0.686839942	0.8451213
rs73762250	G	C	0.222757249	0.307678036	0.469069025
rs7829017	C	T	0.200912176	0.635468856	0.751878269
rs7957385	G	A	0.18171674	1.213438949	0.88095909
rs869408	T	A	0.140348476	0.947198336	0.882206859
rs9423933	G	A	0.095158265	0.161871474	0.556624193
rs9653353	T	C	0.082239905	0.636066608	0.897124635

8. MA-Epilepsy

SNP	EA	OA	Beta	SE	Pval
rs10864152	T	C	0.06907387	0.593998756	0.907425707
rs11232520	G	T	0.122515389	0.698253791	0.860718447
rs12950737	A	G	0.05151215	1.612210082	0.974510917
rs13389386	C	T	0.138719018	0.792021247	0.860965247
rs4721922	G	A	-0.088744692	0.247842281	0.720291512
rs6850727	C	G	0.034262524	0.772569206	0.964626366
rs921038	C	T	-0.050455259	1.157428285	0.965229184

9. MO-Epilepsy

SNP	EA	OA	Beta	SE	Pval
rs12246748	T	C	0.034201932	0.076613457	0.655292445

rs1458265	A	T	-0.063152217	0.132259037	0.633014054
rs1509564	T	G	0.24376331	0.934795324	0.794272576
rs59996663	A	G	0.121650874	0.092666605	0.189257064
rs72829868	A	G	-0.128450177	0.093731693	0.170561191
rs77276355	A	G	0.014155516	0.181173768	0.937722847
rs940659	C	T	-0.291113134	0.195535118	0.136539435
rs9486715	C	A	0.077114585	0.041127244	0.060789373

10. Migraine-Major depressive disorder

SNP	EA	OA	Beta	SE	Pval
rs1038082	C	T	-0.004046732	0.135565511	0.976186087
rs10768192	T	C	-0.008478241	0.093260647	0.927564827
rs10864152	T	C	-0.013792681	0.078158524	0.859924253
rs10870513	T	C	0.011519941	0.158399189	0.942023146
rs10934212	G	C	0.017675936	0.088379679	0.841480581
rs11129828	T	C	0.073859859	0.090904442	0.416504787
rs111503046	C	T	0.193052508	0.077940453	0.013252046
rs11172048	T	C	-0.169159544	0.116729978	0.147294987
rs11200607	T	C	0.061212452	0.069559604	0.37885931
rs117715021	T	C	-0.069371917	0.101389725	0.493842236
rs118113255	T	C	-0.031071561	0.073858629	0.673981716
rs12202086	C	A	0.095648314	0.096273467	0.320463188

rs12779494	T	C	0.024682176	0.078982963	0.754660563
rs12811859	T	G	0.017456947	0.07690493	0.820428513
rs12927082	T	A	0	0.100128033	1
rs12948758	A	G	0.009289853	0.097543453	0.924125712
rs1379828	T	C	-0.027690763	0.093456326	0.767003814
rs141458461	C	G	0.066140618	0.119976005	0.581440341
rs145880920	A	C	0.054146906	0.095846248	0.572117932
rs147855385	T	C	0.042961605	0.0845945	0.611556155
rs186941956	A	G	-0.015880018	0.064108219	0.804361563
rs2001425	T	C	-0.036384217	0.090960543	0.689156517
rs2112563	G	A	-0.050182189	0.080849083	0.534803837
rs2183947	A	G	-0.101794491	0.082405065	0.216721062
rs2462601	C	T	-0.062158544	0.09989766	0.533795756
rs2541318	A	G	-0.118597193	0.118597193	0.317310508
rs2589253	T	C	-0.113217227	0.086681939	0.19151092
rs2976950	A	G	-0.161517029	0.08652698	0.061948151
rs2981889	C	T	0.043455503	0.092836755	0.63972373
rs35231114	A	G	0.194560262	0.092050017	0.034546338
rs35340064	C	A	-0.093043812	0.124058416	0.453254705
rs36010570	C	G	0.068219804	0.064808814	0.292509878
rs37634	T	C	0.144069245	0.088362471	0.103009638
rs4661616	A	T	0.158069445	0.091193911	0.083036439

rs4683465	C	T	0.014501002	0.081568138	0.8588975
rs4910165	G	C	-0.049845393	0.079065107	0.52841016
rs529623	C	T	-0.041825429	0.092015944	0.649436284
rs6112036	A	G	0.282876679	0.097238858	0.003624814
rs62255542	G	C	0.067078957	0.077986104	0.389711951
rs62435157	A	G	0.310862015	0.126695863	0.014143097
rs6713608	T	G	0.241556313	0.094897123	0.010913557
rs6891352	C	T	0.271121177	0.083773398	0.001210631
rs71330635	G	T	-0.07094737	0.093184605	0.446439901
rs72704264	C	G	0.090306605	0.075741024	0.233140619
rs72745900	A	G	0.086693951	0.083422481	0.298704437
rs72829857	G	A	0.265682566	0.078322606	0.000693447
rs7292782	C	A	0.059465355	0.079853477	0.456464678
rs7367621	G	A	-0.125066377	0.090211813	0.165635894
rs73762250	G	C	-0.095859489	0.126781904	0.449590704
rs74522625	G	A	-0.190916467	0.12500483	0.126693208
rs7502966	T	C	0.212233117	0.095504903	0.026268291
rs76710367	T	C	-0.017796679	0.038829117	0.64671298
rs76968534	C	G	0.018931775	0.104913584	0.856798419
rs7829017	C	T	0.007180439	0.080779939	0.929170214
rs7957385	G	A	-0.018043702	0.096984897	0.852408287
rs869408	T	A	0.173343486	0.085124033	0.041713854

rs923688	T	C	0.058175651	0.090714574	0.521324965
rs9305545	G	A	-0.04768969	0.102192193	0.640738382
rs9423933	G	A	0.107914316	0.097422646	0.267994723
rs9486715	C	A	0.027261752	0.089574327	0.760862912
rs9653353	T	C	0.181215558	0.07792269	0.020040893

11. MA-Major depressive disorder

SNP	EA	OA	Beta	SE	Pval
rs10864152	T	C	-0.009281231	0.05259364	0.859924253
rs112276705	G	C	-0.023113842	0.065715827	0.725045156
rs11232520	G	T	0.021551043	0.064653129	0.73888268
rs12568138	A	C	0.03290292	0.065805841	0.617075077
rs12950737	A	G	-0.125017906	0.058602144	0.032897392
rs13389386	C	T	0.106176761	0.061697307	0.085263485
rs142786837	C	T	0.046878042	0.07230342	0.516757536
rs143465900	C	G	-0.059659987	0.069521142	0.390806313
rs151255285	A	C	0.019227072	0.058642569	0.743010825
rs17564412	A	G	0.158932879	0.05959983	0.007660761
rs35356792	G	A	-0.121301494	0.07373228	0.099936554
rs4721922	G	A	-0.080319258	0.06196057	0.194873424
rs653429	A	G	0.079208947	0.064807321	0.221623602
rs6773261	G	A	0.031648221	0.066088932	0.63202881

rs6850727	C	G	-0.018350812	0.056887519	0.747012854
rs7096340	C	G	0.077455232	0.054946874	0.158646437
rs76968534	C	G	0.013377703	0.074134769	0.856798419
rs8180401	A	G	0.164432576	0.060291945	0.006386023
rs921038	C	T	0.057119837	0.067641913	0.398421096

12. MO-Major depressive disorder

SNP	EA	OA	Beta	SE	Pval
rs10774231	C	T	0.021123129	0.054671629	0.699227377
rs11072103	G	T	-0.015237988	0.059779799	0.798798822
rs114974197	A	G	0.012707393	0.06408946	0.842829236
rs116906873	C	T	0.027323856	0.051105731	0.592889505
rs117365086	A	G	-0.085406379	0.068325103	0.211299547
rs12246748	T	C	0.008004391	0.053743768	0.881604
rs1458265	A	T	-0.04814229	0.051845543	0.353111235
rs1509564	T	G	0.067543015	0.060788713	0.266520526
rs151099105	C	T	0.022579548	0.034320913	0.510605773
rs2500283	C	G	-0.001053033	0.054757708	0.984657012
rs3176321	T	C	0.004947549	0.019389045	0.798589937
rs55914748	G	T	-0.007018874	0.061766089	0.90952606
rs59996663	A	G	0.089129712	0.048809128	0.067837165
rs61903808	C	A	-0.006922395	0.044764823	0.877105784

rs67422184	T	C	-0.027628337	0.060628852	0.648608453
rs72829868	A	G	-0.091221023	0.07448322	0.220681075
rs77276355	A	G	-0.009900206	0.061381277	0.871864745
rs78872282	C	A	0.076029414	0.034394259	0.027068656
rs940659	C	T	0.057972723	0.055024958	0.292079176
rs9486715	C	A	0.012516987	0.041127244	0.760862912

13. Migraine-AS

SNP	EA	OA	Beta	SE	Pval
rs1038082	C	T	0.343972191	0.293388045	0.241030964
rs10768192	T	C	0.105978007	0.199238654	0.594784941
rs10864152	T	C	-0.133329247	0.165512168	0.420499161
rs10870513	T	C	0.071999631	0.368638113	0.845148312
rs10909892	A	G	-0.368779476	0.252453064	0.144074642
rs10934212	G	C	0.257907973	0.189614584	0.173776295
rs11129828	T	C	0.102267498	0.196959625	0.603599825
rs111503046	C	T	-0.067148698	0.165473578	0.684891689
rs11172048	T	C	-0.4026195	0.260169357	0.121735853
rs117715021	T	C	-0.054887671	0.248519176	0.825202304
rs118113255	T	C	0.087102246	0.158414026	0.58242965
rs12202086	C	A	0.483867943	0.228180619	0.033959863

rs12779494	T	C	-0.161256882	0.174420709	0.355211323
rs12811859	T	G	-0.277423921	0.221278603	0.209939671
rs12927082	T	A	0.049243295	0.214208332	0.818181088
rs12948758	A	G	-0.230698009	0.238439552	0.333277919
rs141458461	C	G	0.145355544	0.286865704	0.612363637
rs145880920	A	C	-0.146258884	0.229657567	0.524218405
rs147855385	T	C	0.102753529	0.255998016	0.688137366
rs1557343	T	A	-0.119316392	0.184567543	0.517978486
rs186941956	A	G	-0.060579327	0.152918688	0.691991537
rs2001425	T	C	-0.086917853	0.200113195	0.664039067
rs2112563	G	A	-0.124061523	0.171455813	0.469325321
rs2462601	C	T	-0.130976933	0.208675113	0.530226973
rs2541318	A	G	0.011530283	0.240488752	0.961759912
rs2589253	T	C	0.049532537	0.206975243	0.810860374
rs28448367	T	C	0.20267362	0.234014902	0.386450992
rs2976950	A	G	0.036533614	0.188436534	0.846271762
rs2981889	C	T	-0.16394576	0.209376512	0.433616032
rs35231114	A	G	0.129706842	0.2008364	0.518387298
rs35340064	C	A	0.418697154	0.298515564	0.160736927
rs36010570	C	G	0.015008357	0.145308183	0.917735681
rs37634	T	C	0.142148322	0.194013251	0.463757732
rs4661616	A	T	-0.378961362	0.196573541	0.053875646

rs4683465	C	T	-0.222952912	0.175824654	0.204783225
rs4888422	G	A	-0.082126172	0.167985352	0.624920362
rs582161	A	G	0.273721409	0.224763109	0.223291705
rs6112036	A	G	0.139228365	0.212157509	0.511663283
rs62255542	G	C	0.085621108	0.178877218	0.632181568
rs62435157	A	G	-0.167186294	0.258616298	0.517978486
rs6713608	T	G	0.312729155	0.198421257	0.115005765
rs6891352	C	T	0.103574382	0.178208864	0.561107972
rs71330635	G	T	-0.037062059	0.20225295	0.854604945
rs72704264	C	G	-0.087393489	0.170417304	0.608076879
rs72745900	A	G	0.037621903	0.175023636	0.829803751
rs72829857	G	A	-0.024571798	0.165859637	0.882225852
rs7292782	C	A	0.169901016	0.169901016	0.317310508
rs7367621	G	A	0.524868732	0.194775506	0.007044424
rs73762250	G	C	0.332415968	0.267478895	0.213950917
rs7398375	G	C	-0.012078845	0.182692525	0.947285709
rs74522625	G	A	0.321603335	0.321603335	0.317310508
rs7502966	T	C	0.120972877	0.205866123	0.556781431
rs76710367	T	C	0.070493338	0.118336357	0.55137357
rs76968534	C	G	-0.38415726	0.232703063	0.098769727
rs7829017	C	T	-0.098731037	0.172330537	0.566701119
rs7957385	G	A	-0.308998394	0.209758034	0.140719145

rs869408	T	A	-0.173343486	0.184177454	0.346614433
rs923688	T	C	-0.364830354	0.200163681	0.068354887
rs9423933	G	A	0.232315541	0.196344103	0.236727473
rs9486715	C	A	0.258986642	0.186937726	0.165925098
rs9653353	T	C	-0.010872933	0.164906158	0.947430319

14. MA-AS

SNP	EA	OA	Beta	SE	Pval
rs10864152	T	C	-0.089718562	0.111374767	0.420499161
rs112276705	G	C	0.124180252	0.162703323	0.445325526
rs11232520	G	T	0.047412294	0.146547092	0.746294337
rs12568138	A	C	0.007222592	0.140439294	0.958984018
rs12950737	A	G	0.335985623	0.122413366	0.006056977
rs13389386	C	T	0.232441018	0.132003541	0.078260482
rs142786837	C	T	0.021849935	0.172415848	0.899155596
rs143465900	C	G	0.023173714	0.15975071	0.884662135
rs151255285	A	C	0.047106326	0.1408383	0.738024178
rs17564412	A	G	0.092710846	0.127713921	0.467884182
rs35356792	G	A	0.170456347	0.156185583	0.275109862
rs35695296	A	T	0.102934535	0.150541757	0.494126297
rs4721922	G	A	-0.128510812	0.128510812	0.317310508
rs4998284	G	A	0.153365009	0.16004758	0.337938552

rs653429	A	G	-0.015841789	0.164178546	0.92313044
rs6773261	G	A	0.079120552	0.146140313	0.588231029
rs6850727	C	G	-0.200941397	0.1174452	0.087092651
rs7096340	C	G	0.177418821	0.119161895	0.13651663
rs74466079	A	G	-0.126513875	0.16211577	0.435160083
rs755869	G	A	0.011905187	0.165184471	0.942544552
rs76968534	C	G	-0.271455884	0.164434263	0.098769727
rs8180401	A	G	-0.05809951	0.127161192	0.647745403
rs921038	C	T	-0.006012614	0.144302747	0.966764427

15. MO-AS

SNP	EA	OA	Beta	SE	Pval
rs10774231	C	T	-0.29945142	0.116798479	0.01035243
rs11072103	G	T	-0.215676139	0.13596974	0.112692384
rs114974197	A	G	0.343652105	0.144201285	0.017165583
rs116906873	C	T	-0.107524433	0.149016215	0.470563828
rs117365086	A	G	-0.04917337	0.143896808	0.732556638
rs12246748	T	C	-0.110917989	0.116635411	0.34161433
rs1458265	A	T	-0.099987832	0.1126847	0.374904518
rs1509564	T	G	-0.002701721	0.129682588	0.983378607
rs151099105	C	T	0.030407124	0.079178947	0.700955902
rs2318311	G	A	-0.157020792	0.145244233	0.279661048

rs2403304	C	T	0.333399484	0.125686313	0.007986699
rs3176321	T	C	0.018720457	0.04960921	0.70590721
rs55914748	G	T	-0.169154857	0.161434096	0.294718741
rs59996663	A	G	0.055175536	0.102569907	0.590624662
rs61903808	C	A	-0.005537916	0.100605479	0.956101919
rs67422184	T	C	-0.046814683	0.135071872	0.728898677
rs72829868	A	G	-0.024269813	0.158172232	0.87805197
rs7398375	G	C	-0.008421381	0.127373382	0.947285709
rs77276355	A	G	-0.212854427	0.124742595	0.087943082
rs78872282	C	A	-0.108010041	0.078845318	0.170718757
rs940659	C	T	-0.112015093	0.118893212	0.346116483
rs9486715	C	A	0.11891138	0.08583077	0.165925098

16. Migraine-AIS

SNP	EA	OA	Beta	SE	Pval
rs1038082	C	T	0.137588876	0.317668435	0.664926853
rs10768192	T	C	0.084782406	0.216195135	0.694942315
rs10864152	T	C	-0.154784528	0.180837369	0.392035281
rs10870513	T	C	0.220318872	0.397437965	0.579340825
rs10909892	A	G	-0.253690579	0.269778274	0.347029357
rs10934212	G	C	0.138997132	0.203273262	0.494104961

rs11129828	T	C	0.126887451	0.215898051	0.55672072
rs111503046	C	T	-0.002398168	0.173867165	0.988995045
rs11172048	T	C	-0.277975625	0.275997151	0.313853828
rs117715021	T	C	-0.269864382	0.27062671	0.318675643
rs118113255	T	C	0.025468493	0.173695122	0.883425994
rs12202086	C	A	0.359462619	0.245684886	0.143438834
rs12779494	T	C	-0.141511142	0.187584536	0.450617497
rs12811859	T	G	-0.414720453	0.235432885	0.078149916
rs12927082	T	A	-0.084534323	0.241292144	0.726083447
rs12948758	A	G	-0.303468521	0.257019258	0.237712825
rs141458461	C	G	0.39146017	0.308399858	0.204324573
rs145880920	A	C	-0.146258884	0.243349889	0.547824655
rs147855385	T	C	0.093895466	0.274599948	0.732399445
rs1557343	T	A	-0.134230941	0.205075048	0.512760453
rs186941956	A	G	-0.068225261	0.163505367	0.676483718
rs2001425	T	C	0.020213454	0.216283959	0.925539759
rs2112563	G	A	-0.011151598	0.182607411	0.951304495
rs2462601	C	T	-0.190915529	0.228654645	0.403745041
rs2541318	A	G	-0.014824649	0.263549317	0.95514265
rs2589253	T	C	-0.019459211	0.201668185	0.92313044
rs28448367	T	C	0.104470938	0.254909089	0.681926208
rs2976950	A	G	0.084604158	0.196127822	0.666197507

rs2981889	C	T	-0.260733015	0.227153763	0.251040366
rs35231114	A	G	0.121338658	0.217572767	0.577054511
rs35340064	C	A	0.395436201	0.324361067	0.222797319
rs36010570	C	G	0.154176758	0.156223352	0.323691878
rs37634	T	C	0.155594785	0.209380637	0.457409476
rs4661616	A	T	-0.470155273	0.212785792	0.027138228
rs4683465	C	T	-0.21207716	0.188513031	0.260589034
rs4888422	G	A	-0.126922266	0.186650391	0.496504461
rs582161	A	G	0.275946787	0.242566127	0.255281414
rs6112036	A	G	0.207737561	0.227627328	0.361441717
rs62255542	G	C	0.110707547	0.192511153	0.565243401
rs62435157	A	G	-0.047021145	0.282126871	0.867632335
rs6713608	T	G	0.284691369	0.215675279	0.186835018
rs6891352	C	T	0.223903444	0.194963543	0.250788
rs71330635	G	T	0.018001571	0.221313437	0.935171792
rs72704264	C	G	-0.132546792	0.184982885	0.473660797
rs72745900	A	G	-0.047436313	0.186473781	0.799197393
rs72829857	G	A	0.082929818	0.18121701	0.647220357
rs7292782	C	A	0.261647564	0.185192107	0.157701587
rs7367621	G	A	0.584326518	0.211177654	0.005657644
rs73762250	G	C	0.482389684	0.292216828	0.098780717
rs7398375	G	C	-0.092101191	0.19477137	0.636307201

rs74522625	G	A	0.386378565	0.343195078	0.260238408
rs7502966	T	C	0.241945753	0.224967104	0.282163597
rs76710367	T	C	0.051078779	0.127119134	0.68781784
rs76968534	C	G	-0.459095534	0.249268366	0.065508485
rs7829017	C	T	-0.165150098	0.186691415	0.376364106
rs7957385	G	A	-0.27291099	0.227801736	0.230909308
rs869408	T	A	-0.119173647	0.212036229	0.57408618
rs923688	T	C	-0.374690634	0.217912184	0.085531188
rs9423933	G	A	0.380697726	0.215828632	0.077750688
rs9486715	C	A	0.426451688	0.204463138	0.037004512
rs9653353	T	C	-0.052552512	0.181215558	0.771816238

17. MA-AIS

SNP	EA	OA	Beta	SE	Pval
rs10864152	T	C	-0.104156032	0.121687245	0.392035281
rs112276705	G	C	0.173580425	0.170861149	0.309669808
rs11232520	G	T	0.010057153	0.149420564	0.946336753
rs12568138	A	C	0.020865267	0.150871927	0.890005007
rs12950737	A	G	0.35942648	0.132831525	0.006812318
rs13389386	C	T	0.304182073	0.144916931	0.035816028
rs142786837	C	T	0.057207102	0.189101253	0.762254921
rs143465900	C	G	0.034514042	0.176021616	0.844548778

rs151255285	A	C	0.088925207	0.153335897	0.561956898
rs17564412	A	G	0.157040821	0.140012298	0.262023354
rs35356792	G	A	0.245774268	0.170456347	0.149341741
rs35695296	A	T	0.263769746	0.172415346	0.126053677
rs4721922	G	A	-0.143427246	0.138837574	0.301576826
rs4998284	G	A	0.116276739	0.172076208	0.499213257
rs653429	A	G	-0.011521301	0.174259684	0.947285709
rs6773261	G	A	0.057711461	0.153586953	0.70709713
rs6850727	C	G	-0.2055291	0.128455687	0.109598583
rs7096340	C	G	0.21449141	0.125782	0.088145355
rs74466079	A	G	-0.047045361	0.177373725	0.790829994
rs755869	G	A	0.156255581	0.178577806	0.381573906
rs76968534	C	G	-0.324409291	0.176139752	0.065508485
rs8180401	A	G	0.005481086	0.140315798	0.968840559
rs921038	C	T	0.045094608	0.15783113	0.775096962

18. MO-AIS

SNP	EA	OA	Beta	SE	Pval
rs10774231	C	T	-0.284540976	0.130466386	0.029186939
rs11072103	G	T	-0.264906562	0.148863422	0.075153311
rs114974197	A	G	0.379564302	0.155803687	0.014843698
rs116906873	C	T	0.076405597	0.165208129	0.643736527

rs117365086	A	G	-0.083335921	0.156319554	0.593955649
rs12246748	T	C	-0.102913598	0.126926771	0.417474325
rs1458265	A	T	-0.190453014	0.123794459	0.123935806
rs1509564	T	G	-0.014859463	0.141840331	0.916564739
rs151099105	C	T	0.109586072	0.085501221	0.199951365
rs2318311	G	A	-0.099446502	0.157020792	0.52651599
rs2403304	C	T	0.22491235	0.136270424	0.098843689
rs3176321	T	C	0.020057632	0.053487019	0.707660467
rs55914748	G	T	-0.061064202	0.17126052	0.721423191
rs59996663	A	G	0.077811653	0.111765829	0.486302002
rs61903808	C	A	-0.030458539	0.109835339	0.781541372
rs67422184	T	C	-0.007674538	0.146583679	0.958245016
rs72829868	A	G	0.057745418	0.170725584	0.735185881
rs7398375	G	C	-0.064213027	0.135794763	0.636307201
rs77276355	A	G	-0.238594963	0.136622842	0.080745497
rs78872282	C	A	-0.110825945	0.086890759	0.202145425
rs940659	C	T	-0.015721416	0.129701686	0.903523024
rs9486715	C	A	0.195801445	0.093877405	0.037004512

19. Migraine-LAS

SNP	EA	OA	Beta	SE	Pval
rs1038082	C	T	0.694014479	0.803276234	0.38759896

rs10768192	T	C	-0.337010064	0.538368278	0.531325271
rs10864152	T	C	0.174707289	0.447495862	0.696232686
rs10870513	T	C	1.511992259	0.976315001	0.121460452
rs10909892	A	G	-0.690533381	0.697958471	0.322486205
rs10934212	G	C	0.851658726	0.530278075	0.108260594
rs11129828	T	C	0.138250506	0.537851284	0.797146155
rs111503046	C	T	-0.607935537	0.447258295	0.174067594
rs11172048	T	C	0.271050965	0.691476733	0.69506667
rs117715021	T	C	0.635019859	0.682284242	0.351995184
rs118113255	T	C	-0.314790573	0.482882626	0.514466502
rs12202086	C	A	1.314070305	0.607022962	0.030404646
rs12779494	T	C	0.054300787	0.465670384	0.907170877
rs12811859	T	G	-1.176220807	0.586459071	0.044895575
rs12927082	T	A	0.210104724	0.60323036	0.727615405
rs12948758	A	G	-0.066577278	0.606937043	0.912652156
rs141458461	C	G	1.495085598	0.802147263	0.062342173
rs145880920	A	C	-0.510972528	0.667189464	0.443760675
rs147855385	T	C	0.577102793	0.652396327	0.376378194
rs1557343	T	A	0.365406449	0.49963739	0.464569499
rs186941956	A	G	-0.14233201	0.421114542	0.735371608
rs2001425	T	C	-0.507357697	0.539699224	0.347179139
rs2112563	G	A	0.404245413	0.457215502	0.37661722

rs2462601	C	T	-0.213115009	0.559426898	0.703238583
rs2541318	A	G	-0.121891559	0.658873294	0.853229036
rs2589253	T	C	0.357341872	0.500632424	0.47536266
rs28448367	T	C	1.460503717	0.641451561	0.022793813
rs2976950	A	G	-0.048070544	0.488396732	0.921594668
rs2981889	C	T	0.377272772	0.576773034	0.513041204
rs35231114	A	G	0.330543242	0.539747825	0.540271091
rs35340064	C	A	-0.54275557	0.827056107	0.511663283
rs36010570	C	G	0.057986834	0.396357062	0.883684973
rs37634	T	C	-0.315031417	0.530174823	0.552376414
rs4661616	A	T	-0.986920767	0.530951213	0.063058523
rs4683465	C	T	-0.094256515	0.476720453	0.843265212
rs4888422	G	A	-0.970582032	0.464759473	0.036765967
rs582161	A	G	0.558569705	0.605302629	0.356114507
rs6112036	A	G	0.139228365	0.563543384	0.8048627
rs62255542	G	C	0.533904868	0.490276278	0.276159281
rs62435157	A	G	-1.030546764	0.705317177	0.143984951
rs6713608	T	G	1.099943924	0.526247681	0.036603076
rs6891352	C	T	0.059402955	0.485885706	0.902695443
rs71330635	G	T	0.674529471	0.531575815	0.204468064
rs72704264	C	G	-0.429684655	0.463185493	0.353577241
rs72745900	A	G	0.266624792	0.475998862	0.575385683

rs72829857	G	A	-0.06450097	0.445363839	0.884847001
rs7292782	C	A	-0.380578275	0.457033732	0.405006185
rs7367621	G	A	0.711443163	0.520768195	0.171894482
rs73762250	G	C	-1.015801354	0.714307802	0.155003631
rs7398375	G	C	-0.679435012	0.486173498	0.1622586
rs74522625	G	A	0.071593675	0.92049011	0.938004824
rs7502966	T	C	-0.602742052	0.553928435	0.276540986
rs76710367	T	C	0.469647418	0.294454139	0.110717502
rs76968534	C	G	-0.671289175	0.659456816	0.308705243
rs7829017	C	T	-0.050263073	0.463138319	0.91357756
rs7957385	G	A	-0.315764782	0.575142996	0.582991994
rs869408	T	A	0.489076265	0.492171684	0.320363736
rs923688	T	C	-0.296794423	0.554147727	0.592243885
rs9423933	G	A	0.469127513	0.529079911	0.375248219
rs9486715	C	A	0.549129571	0.500447872	0.272520643
rs9653353	T	C	0.146784602	0.451226739	0.744953119

20. MA-LAS

SNP	EA	OA	Beta	SE	Pval
rs10864152	T	C	0.117562254	0.30112437	0.696232686
rs112276705	G	C	0.600959904	0.428285905	0.160564779
rs11232520	G	T	0.152294037	0.382171828	0.690264429

rs12568138	A	C	-0.958999751	0.371562247	0.009851643
rs12950737	A	G	0.802198232	0.325567464	0.013739611
rs13389386	C	T	0.411793656	0.354400812	0.245258489
rs142786837	C	T	-0.170826762	0.522809345	0.743858704
rs143465900	C	G	0.111431051	0.44424503	0.801943684
rs151255285	A	C	0.732070756	0.370121131	0.047937498
rs17564412	A	G	0.396386169	0.349084717	0.256165314
rs35356792	G	A	-0.004756921	0.440808042	0.991389902
rs35695296	A	T	0.142821667	0.387291188	0.712298598
rs4721922	G	A	-0.152606589	0.343077972	0.656452692
rs4998284	G	A	0.793889457	0.407970971	0.051661233
rs653429	A	G	0.373002134	0.437809455	0.394228698
rs6773261	G	A	-0.252254936	0.396533589	0.524678506
rs6850727	C	G	-0.106434712	0.317469056	0.737428878
rs7096340	C	G	0.115851842	0.312468968	0.710814277
rs74466079	A	G	0.127785371	0.472996599	0.787036172
rs755869	G	A	0.355667464	0.453885258	0.433271007
rs76968534	C	G	-0.474351042	0.465989978	0.308705243
rs8180401	A	G	-0.513029637	0.347500844	0.139852455
rs921038	C	T	-0.242007732	0.393826247	0.538882452

21. MO-LAS

SNP	EA	OA	Beta	SE	Pval
rs10774231	C	T	-0.250992476	0.320574549	0.433658978
rs11072103	G	T	0.365711714	0.379777549	0.33556611
rs114974197	A	G	0.873495141	0.392271695	0.02596328
rs116906873	C	T	-0.099175477	0.425796756	0.815825539
rs117365086	A	G	-0.005176144	0.414091535	0.990026703
rs12246748	T	C	0.177240086	0.313314733	0.571602202
rs1458265	A	T	0.062426266	0.317950726	0.844344498
rs1509564	T	G	-0.016210324	0.348521956	0.962902466
rs151099105	C	T	0.246267601	0.233020933	0.290581171
rs2318311	G	A	0.189733457	0.389934967	0.626557998
rs2403304	C	T	0.336045512	0.338691539	0.321106069
rs3176321	T	C	-0.02674351	0.141205731	0.849784074
rs55914748	G	T	-0.910347926	0.437977722	0.037660972
rs59996663	A	G	0.275170301	0.284366224	0.333213356
rs61903808	C	A	0.258897585	0.278741785	0.352988625
rs67422184	T	C	0.262469206	0.357633479	0.463006286
rs72829868	A	G	0.340614277	0.438530421	0.437325426
rs7398375	G	C	-0.47370266	0.33896057	0.1622586
rs77276355	A	G	-0.56827182	0.342547125	0.097123946
rs78872282	C	A	-0.336299419	0.207170097	0.104525082
rs940659	C	T	-0.34783634	0.322289038	0.280468143

rs9486715	C	A	0.252127888	0.229776125	0.272520643

22. Migraine-CES

SNP	EA	OA	Beta	SE	Pval
rs1038082	C	T	-0.070817804	0.619149944	0.908937293
rs10768192	T	C	-0.019076041	0.415433789	0.963375315
rs10864152	T	C	-0.514926746	0.343284497	0.133614403
rs10870513	T	C	0.347038223	0.839515702	0.679328904
rs10909892	A	G	-0.444267892	0.522231339	0.394929966
rs10934212	G	C	-0.041779485	0.417794847	0.920344325
rs11129828	T	C	0.621180356	0.416645361	0.135985364
rs111503046	C	T	0.074343202	0.352530667	0.832977513
rs11172048	T	C	0.435264324	0.540123457	0.420323113
rs117715021	T	C	-0.336186984	0.529056161	0.525137128
rs118113255	T	C	0.002546849	0.374386846	0.994572256
rs12202086	C	A	0.827701752	0.5382562	0.124110534
rs12779494	T	C	-0.199102885	0.363650724	0.584027501
rs12811859	T	G	-0.386883699	0.482189196	0.422351489
rs12927082	T	A	0.144446998	0.480942845	0.763916836
rs12948758	A	G	-0.535714839	0.467589252	0.251921223
rs141458461	C	G	0.604494486	0.617568794	0.327664286
rs145880920	A	C	-0.121986133	0.521552958	0.81507021

rs147855385	T	C	0.736990827	0.55407183	0.183473502
rs1557343	T	A	-0.326255758	0.376592361	0.386305582
rs186941956	A	G	-0.853403911	0.32406999	0.008453626
rs2001425	T	C	0.652894567	0.414375809	0.115115766
rs2112563	G	A	-0.366608771	0.361032972	0.309894221
rs2462601	C	T	-0.277493501	0.437329758	0.525742976
rs2541318	A	G	-0.345908479	0.512273986	0.499522562
rs2589253	T	C	-0.04245646	0.390953236	0.913521914
rs28448367	T	C	-0.127454545	0.495192247	0.796882379
rs2976950	A	G	0.296114554	0.380718712	0.436700031
rs2981889	C	T	-0.148143759	0.438505526	0.735485394
rs35231114	A	G	-0.135982979	0.416317121	0.743945356
rs35340064	C	A	0.032306879	0.624168906	0.95872006
rs36010570	C	G	0.630350991	0.310400109	0.04227841
rs37634	T	C	0.505202821	0.405314811	0.212600887
rs4661616	A	T	-0.178334759	0.411385864	0.664653355
rs4683465	C	T	-0.558288592	0.369775561	0.131093464
rs4888422	G	A	-0.039196582	0.360235254	0.913354548
rs582161	A	G	0.778882059	0.460653104	0.090870946
rs6112036	A	G	0.15248821	0.435364889	0.726148229
rs62255542	G	C	-0.063806813	0.401383026	0.873694565
rs62435157	A	G	0.576009028	0.564253742	0.307333409

rs6713608	T	G	1.173273519	0.407626278	0.00399816
rs6891352	C	T	0.3122463	0.377741865	0.408456501
rs71330635	G	T	-0.49345484	0.434155546	0.255711661
rs72704264	C	G	0.064088559	0.356856748	0.857473014
rs72745900	A	G	0.037621903	0.368040357	0.918580289
rs72829857	G	A	0.144359313	0.348612384	0.678803131
rs7292782	C	A	0.10533863	0.353394113	0.765644454
rs7367621	G	A	0.885715985	0.403902891	0.02831506
rs73762250	G	C	0.219549151	0.567426327	0.698814708
rs7398375	G	C	0.469565086	0.37595404	0.211666543
rs74522625	G	A	-0.938672631	0.637524632	0.140920783
rs7502966	T	C	0.106116558	0.428710896	0.804502138
rs76710367	T	C	0.38251303	0.263945547	0.147278413
rs76968534	C	G	0.457517887	0.526934394	0.385250114
rs7829017	C	T	0.445187221	0.355431733	0.210378533
rs7957385	G	A	-1.019469154	0.43755977	0.019811601
rs869408	T	A	0.05107442	0.388475134	0.895400271
rs923688	T	C	-0.824319394	0.424978061	0.052419179
rs9423933	G	A	0.212831012	0.412172735	0.605600458
rs9486715	C	A	0.960003116	0.385559061	0.012777941
rs9653353	T	C	-0.829967254	0.387801294	0.032339664

23. MA-CES

SNP	EA	OA	Beta	SE	Pval
rs10864152	T	C	-0.346499275	0.230999516	0.133614403
rs112276705	G	C	0.20938422	0.349426913	0.549024994
rs11232520	G	T	-0.004310209	0.288783975	0.988091717
rs12568138	A	C	0.12679662	0.297731304	0.670198196
rs12950737	A	G	0.402401386	0.252640352	0.111208767
rs13389386	C	T	0.595450756	0.27405083	0.029797266
rs142786837	C	T	0.883929174	0.445738667	0.047360063
rs143465900	C	G	-0.434383875	0.337744557	0.198397212
rs151255285	A	C	0.319169391	0.327340896	0.32954205
rs17564412	A	G	-0.11825363	0.267726219	0.658709123
rs35356792	G	A	0.434465481	0.333777313	0.193031713
rs35695296	A	T	0.146681712	0.298510151	0.623158195
rs4721922	G	A	-0.169817859	0.268495804	0.527074167
rs4998284	G	A	0.131312524	0.35016673	0.707660467
rs653429	A	G	-0.076328622	0.331237417	0.817753936
rs6773261	G	A	0.212229245	0.304381417	0.485647788
rs6850727	C	G	-0.199106315	0.25048859	0.426688083
rs7096340	C	G	-0.123133958	0.23964781	0.607383329
rs74466079	A	G	0.005085985	0.34775422	0.988331172
rs755869	G	A	-0.38691858	0.345250426	0.262419987

rs76968534	C	G	0.323294482	0.372346059	0.385250114
rs8180401	A	G	0.026309212	0.272958076	0.923214374
rs921038	C	T	0.432908241	0.300630723	0.149867399

24. MO-CES

SNP	EA	OA	Beta	SE	Pval
rs10774231	C	T	-0.641149098	0.243537254	0.008472084
rs11072103	G	T	-0.622413205	0.291866079	0.032963291
rs114974197	A	G	0.04861959	0.318789814	0.878782339
rs116906873	C	T	0.03415482	0.328392269	0.917164457
rs117365086	A	G	-0.160978084	0.312639109	0.606622438
rs12246748	T	C	-0.138361616	0.245849152	0.573577415
rs1458265	A	T	0.115858917	0.243885665	0.634748321
rs1509564	T	G	-0.068893875	0.268821199	0.797733553
rs151099105	C	T	0.229107144	0.17009926	0.178011586
rs2318311	G	A	-0.228988655	0.300956518	0.446734979
rs2403304	C	T	0.31487729	0.260633724	0.227000405
rs3176321	T	C	0.078091048	0.117002855	0.504498421
rs55914748	G	T	0.143886912	0.387441831	0.710356894
rs59996663	A	G	0.251826805	0.217165251	0.246207986
rs61903808	C	A	-0.461031529	0.221516651	0.037411029
rs67422184	T	C	-0.388331632	0.288562636	0.178384871

rs72829868	A	G	0.57829107	0.338103607	0.087192663
rs7398375	G	C	0.327381172	0.262115472	0.211666543
rs77276355	A	G	-0.441549184	0.265325519	0.096076643
rs78872282	C	A	-0.330466475	0.164529261	0.044584087
rs940659	C	T	-0.28102032	0.253507841	0.267634228
rs9486715	C	A	0.440776768	0.177025964	0.012777941

25. Migraine-SVS

SNP	EA	OA	Beta	SE	Pval
rs1038082	C	T	-0.483584433	0.75673882	0.52279858
rs10768192	T	C	0.464183673	0.502335755	0.355459954
rs10864152	T	C	-0.617605591	0.415312941	0.136992397
rs10870513	T	C	-0.050399742	0.925915259	0.956590707
rs10909892	A	G	-1.002387166	0.610094905	0.100382514
rs10934212	G	C	0.252283811	0.482070977	0.600742308
rs11129828	T	C	0.657163365	0.501868276	0.190387393
rs111503046	C	T	-0.509610657	0.416082113	0.220656667
rs11172048	T	C	-0.901194998	0.63113327	0.153320734
rs117715021	T	C	0.319415751	0.643405475	0.619580132
rs118113255	T	C	0.761507939	0.418192654	0.068614448
rs12202086	C	A	0.259438238	0.593269609	0.661892038
rs12779494	T	C	-0.519971171	0.42946986	0.225999679

rs12811859	T	G	0.292993631	0.595895258	0.622941011
rs12927082	T	A	1.185121959	0.563015003	0.035295115
rs12948758	A	G	-0.605388734	0.572874249	0.290622615
rs141458461	C	G	0.653715411	0.73216126	0.37193368
rs145880920	A	C	0.125720403	0.596860724	0.833171257
rs147855385	T	C	0.543442155	0.550971508	0.323969042
rs1557343	T	A	0.333713033	0.464215336	0.472217632
rs186941956	A	G	0.305249228	0.383473019	0.426024904
rs2001425	T	C	-0.07478978	0.505336352	0.882342768
rs2112563	G	A	-0.086424881	0.430730458	0.84097441
rs2462601	C	T	-0.268613709	0.521687782	0.606627361
rs2541318	A	G	0.817002884	0.61110498	0.181246393
rs2589253	T	C	0.613849651	0.461714003	0.183682434
rs28448367	T	C	0.240283158	0.597573767	0.687611977
rs2976950	A	G	-0.180745247	0.459554405	0.694093901
rs2981889	C	T	-0.086911005	0.525416531	0.868618502
rs35231114	A	G	0.347279608	0.502091	0.489146685
rs35340064	C	A	0.04006053	0.740473671	0.956854519
rs36010570	C	G	0.921649555	0.35883617	0.010215708
rs37634	T	C	-0.232431716	0.487914511	0.633805139
rs4661616	A	T	-0.535004276	0.492447118	0.277293326
rs4683465	C	T	-0.581852721	0.442280573	0.188317142

rs4888422	G	A	0.029864063	0.431162403	0.944779426
rs582161	A	G	0.68764159	0.569696592	0.227420109
rs6112036	A	G	0.446414759	0.528183799	0.398005722
rs62255542	G	C	0.359390509	0.447738403	0.422159913
rs62435157	A	G	-1.485084501	0.662214461	0.024922391
rs6713608	T	G	-0.129405168	0.489582884	0.791535523
rs6891352	C	T	0.013708374	0.449330041	0.975661535
rs71330635	G	T	-0.50933858	0.523104487	0.330213359
rs72704264	C	G	-0.882674241	0.435510888	0.042687387
rs72745900	A	G	0.073608071	0.441648428	0.867632335
rs72829857	G	A	0.568222829	0.420792041	0.176898944
rs7292782	C	A	0.011893071	0.428150559	0.97783939
rs7367621	G	A	0.56792437	0.479762825	0.23650781
rs73762250	G	C	0.858097035	0.680293145	0.207177863
rs7398375	G	C	0.003019711	0.448427108	0.994627081
rs74522625	G	A	1.765977322	0.840941582	0.035728841
rs7502966	T	C	0.570907084	0.517848805	0.270262167
rs76710367	T	C	-0.287751494	0.283360105	0.309868712
rs76968534	C	G	-1.101198224	0.611338555	0.071657132
rs7829017	C	T	-0.951408174	0.429031233	0.026583945
rs7957385	G	A	-0.311253857	0.543566518	0.566906005
rs869408	T	A	-0.054169839	0.456574361	0.905557351

rs923688	T	C	0.248479052	0.533441139	0.641355754
rs9423933	G	A	0.593528738	0.509595381	0.244138067
rs9486715	C	A	1.006737547	0.469291584	0.031934605
rs9653353	T	C	0.590762718	0.422232249	0.161770516

26. MA-SVS

SNP	EA	OA	Beta	SE	Pval
rs10864152	T	C	-0.41559288	0.279468165	0.136992397
rs112276705	G	C	0.870168187	0.401093149	0.030045387
rs11232520	G	T	0.397975926	0.353437104	0.260158637
rs12568138	A	C	0.100313782	0.352302001	0.775844844
rs12950737	A	G	0.27087213	0.303428877	0.372015781
rs13389386	C	T	0.375923128	0.324269568	0.246337167
rs142786837	C	T	0.117195104	0.459245899	0.798576114
rs143465900	C	G	0.702114232	0.466432629	0.132250714
rs151255285	A	C	0.078830994	0.366275716	0.829593384
rs17564412	A	G	0.022704697	0.32638002	0.944539722
rs35356792	G	A	0.543874671	0.402752672	0.17688973
rs35695296	A	T	0.119661397	0.360270872	0.73978139
rs4721922	G	A	0.240957773	0.330456374	0.465899719
rs4998284	G	A	-0.679283361	0.414987671	0.10165638
rs653429	A	G	-0.309634976	0.387403761	0.424141693

rs6773261	G	A	-0.364885368	0.386294459	0.344874203
rs6850727	C	G	-0.300035784	0.289942837	0.300757593
rs7096340	C	G	-0.057594916	0.293932674	0.844652475
rs74466079	A	G	-0.363012175	0.414507772	0.381156783
rs755869	G	A	0.330368942	0.418169697	0.429506985
rs76968534	C	G	-0.778136375	0.431988317	0.071657132
rs8180401	A	G	-0.571129148	0.323384066	0.077378789
rs921038	C	T	0.704979046	0.363763175	0.052621206

27. MO-SVS

SNP	EA	OA	Beta	SE	Pval
rs10774231	C	T	-0.544231211	0.296966346	0.066856723
rs11072103	G	T	-0.600142299	0.355162337	0.091072161
rs114974197	A	G	0.443653762	0.379011807	0.241778122
rs116906873	C	T	-0.226180808	0.391135939	0.563084212
rs117365086	A	G	-0.259324824	0.374752839	0.488944268
rs12246748	T	C	-0.202396743	0.290445044	0.485896425
rs1458265	A	T	-0.807309163	0.293615063	0.005967724
rs1509564	T	G	-0.0918585	0.328259052	0.779603798
rs151099105	C	T	0.184851231	0.207430779	0.372850534
rs2318311	G	A	-0.26170132	0.363764835	0.47187943
rs2403304	C	T	-0.056889594	0.316200304	0.857218264

rs3176321	T	C	-0.111921588	0.127833976	0.381289394
rs55914748	G	T	0.106686881	0.392355043	0.785688044
rs59996663	A	G	-0.019099224	0.265267	0.942601908
rs61903808	C	A	0.093683084	0.257513106	0.716007898
rs67422184	T	C	-0.175746924	0.341516949	0.606827853
rs72829868	A	G	-0.228471002	0.403381036	0.571128663
rs7398375	G	C	0.002105345	0.312643756	0.994627081
rs77276355	A	G	0.059401236	0.31779661	0.851726582
rs78872282	C	A	-0.093729384	0.205762145	0.648733045
rs940659	C	T	0.112015093	0.298706913	0.707660467
rs9486715	C	A	0.462234461	0.215470996	0.031934605

28. Migraine-insomnia

SNP	EA	OA	Beta	SE	Pval
rs1038082	C	T	-0.023292785	0.052627948	0.65805982
rs10768192	T	C	0.02980144	0.036039729	0.408290792
rs10864152	T	C	0.007448339	0.030648716	0.807987684
rs10870513	T	C	-0.035079948	0.061273126	0.566971308
rs10909892	A	G	0.040898139	0.039124037	0.295863286
rs10934212	G	C	0.026244346	0.034551714	0.447513385
rs11129828	T	C	0.055001165	0.035088168	0.116994794
rs111503046	C	T	0.023810449	0.030388503	0.433313145

rs11172048	T	C	0.084129274	0.047676282	0.077632052
rs11200607	T	C	0.007323486	0.0269933	0.786154516
rs117715021	T	C	0.064271481	0.037991874	0.090700035
rs118113255	T	C	0.02464255	0.028850607	0.39302565
rs12202086	C	A	0.095788974	0.036428129	0.00855022
rs12779494	T	C	-0.015040594	0.030715158	0.624360461
rs12811859	T	G	-0.021659071	0.028445199	0.446399384
rs12927082	T	A	-0.013653935	0.038107826	0.720120775
rs12948758	A	G	-0.042484819	0.03650556	0.244508979
rs141458461	C	G	0.011391414	0.04480973	0.799327594
rs145880920	A	C	-0.064652651	0.036162727	0.073804142
rs147855385	T	C	-0.030985548	0.032292024	0.33728585
rs1557343	T	A	0.061817823	0.033544498	0.065349419
rs186941956	A	G	0.058594207	0.023781679	0.013745819
rs2001425	T	C	0.013676645	0.034870432	0.694900655
rs2112563	G	A	-0.024101948	0.031223916	0.440169724
rs2183947	A	G	-0.038449072	0.032323629	0.234241566
rs2462601	C	T	-0.000851394	0.037276701	0.981778016
rs2541318	A	G	0.157128597	0.046267565	0.000683571
rs2589253	T	C	0.043944559	0.033722105	0.192527624
rs28448367	T	C	0.037453876	0.04019394	0.35142494
rs2976950	A	G	-0.051933686	0.032731234	0.112587272

rs2981889	C	T	-0.049021165	0.034653591	0.157184152
rs35231114	A	G	-0.04133778	0.035139048	0.239432756
rs35340064	C	A	0.02868166	0.047534921	0.546255374
rs36010570	C	G	0.007110004	0.025459563	0.780040269
rs37634	T	C	0.015121969	0.033846476	0.655033049
rs4661616	A	T	-0.025104062	0.035259619	0.476478585
rs4683465	C	T	0.015288751	0.032063891	0.63348927
rs4888422	G	A	-0.022075702	0.033312615	0.50753364
rs4910165	G	C	0.039598212	0.030999022	0.20146037
rs529623	C	T	-0.025665756	0.035034443	0.463810735
rs582161	A	G	-0.013458414	0.038797673	0.728676099
rs6112036	A	G	0.105099957	0.037962934	0.005631693
rs62111968	T	C	0.048451631	0.032411759	0.134946246
rs62255542	G	C	0.045000327	0.030269461	0.137105242
rs62435157	A	G	0.07560765	0.050529053	0.134570196
rs6713608	T	G	-0.055182893	0.036544235	0.131035759
rs6891352	C	T	-0.016052049	0.032555104	0.621960868
rs71330635	G	T	-0.075486095	0.036043699	0.036233818
rs72704264	C	G	-0.001254373	0.029338286	0.965896431
rs72745900	A	G	-0.037382268	0.032098026	0.244169507
rs72829857	G	A	0.002600479	0.03053522	0.932131583
rs7292782	C	A	0.086341997	0.030883587	0.005178354

rs7367621	G	A	0.054914186	0.0349376	0.116001814
rs73762250	G	C	-0.120731161	0.049435202	0.01459764
rs7398375	G	C	0.009972113	0.029416818	0.734614556
rs74522625	G	A	0.081045859	0.047266599	0.086408699
rs7502966	T	C	-0.023551933	0.036647141	0.520439753
rs76710367	T	C	0.009372309	0.01413648	0.507338583
rs76968534	C	G	0.045216572	0.040635319	0.265819826
rs7829017	C	T	-0.008203454	0.031674891	0.795643588
rs7957385	G	A	0.001189662	0.037934402	0.974981621
rs869408	T	A	0.034465483	0.03296374	0.29576542
rs923688	T	C	0.024071901	0.035410434	0.496633109
rs9266329	A	G	-0.049680752	0.036775633	0.176722683
rs9305545	G	A	-0.023745548	0.039783591	0.550595583
rs9423933	G	A	-0.018153136	0.036635561	0.620243066
rs9486715	C	A	0.022460178	0.034933988	0.520268227
rs9653353	T	C	-0.013013796	0.030352881	0.668105213

29. MA-insomnia

SNP	EA	OA	Beta	SE	Pval
rs10864152	T	C	0.00501206	0.020623822	0.807987684
rs11148391	A	G	-0.002183645	0.024933615	0.930211806
rs112276705	G	C	-0.004869452	0.024809537	0.844396185

rs11232520	G	T	0.020643888	0.0253469	0.415384832
rs12568138	A	C	0.008985707	0.025468225	0.724223145
rs12950737	A	G	-0.031637735	0.022658454	0.162627685
rs13389386	C	T	-0.051768632	0.024250485	0.032781744
rs142786837	C	T	0.033466313	0.028123408	0.234053976
rs143465900	C	G	-0.019226146	0.026055587	0.460581564
rs151255285	A	C	-0.008559123	0.022451404	0.703033428
rs17564412	A	G	0.007282371	0.02308396	0.752402396
rs35356792	G	A	-0.010106714	0.028876812	0.726343128
rs35695296	A	T	0.035417457	0.026939383	0.188607621
rs4721922	G	A	-0.044167789	0.023970135	0.065384883
rs4998284	G	A	0.016697206	0.021311655	0.433346657
rs62111968	T	C	0.030878781	0.020656387	0.134946246
rs653429	A	G	0.027089028	0.024709159	0.272940838
rs6773261	G	A	0.053670821	0.025662984	0.036494815
rs6850727	C	G	0.031005716	0.021057924	0.140912528
rs7096340	C	G	0.007214127	0.020386151	0.723433364
rs755869	G	A	0.00271154	0.025927563	0.916707991
rs76968534	C	G	0.031951249	0.028714013	0.265819826
rs8180401	A	G	0.025954038	0.023202971	0.263325645
rs921038	C	T	0.002857209	0.026527054	0.914226321

30. MO-insomnia

SNP	EA	OA	Beta	SE	Pval
rs10774231	C	T	0.017211126	0.021094178	0.414546872
rs11072103	G	T	0.033196543	0.023049918	0.149810204
rs114974197	A	G	0.00934673	0.024481732	0.702621708
rs116906873	C	T	-0.009301142	0.018618425	0.617380335
rs11955425	T	A	0.01171637	0.026799713	0.661978889
rs12246748	T	C	-0.018066025	0.020728171	0.383443716
rs1458265	A	T	0.052461499	0.020363977	0.009989537
rs1509564	T	G	0.064423743	0.023802429	0.006797533
rs151099105	C	T	-0.007370506	0.013284933	0.579030151
rs2318311	G	A	-0.028144407	0.0257607	0.274599037
rs2500283	C	G	0.027577351	0.020897858	0.186960016
rs3176321	T	C	0.002959878	0.007331292	0.686409715
rs55914748	G	T	-0.005526991	0.02437332	0.820607272
rs59996663	A	G	0.040960549	0.01896751	0.030810533
rs61903808	C	A	0.007428884	0.017216459	0.666105613
rs67422184	T	C	-0.037423351	0.022947713	0.102930508
rs72829868	A	G	-0.00059782	0.029050046	0.98358152
rs7398375	G	C	0.006952566	0.02050943	0.734614556
rs77276355	A	G	-0.019375594	0.024131356	0.422019881
rs78872282	C	A	0.005299069	0.013456764	0.693739922

rs940659	C	T	0.031444307	0.021918209	0.151395342
rs9486715	C	A	0.010312388	0.016039625	0.520268227

Supplementary Figure 1:

Scatter plot

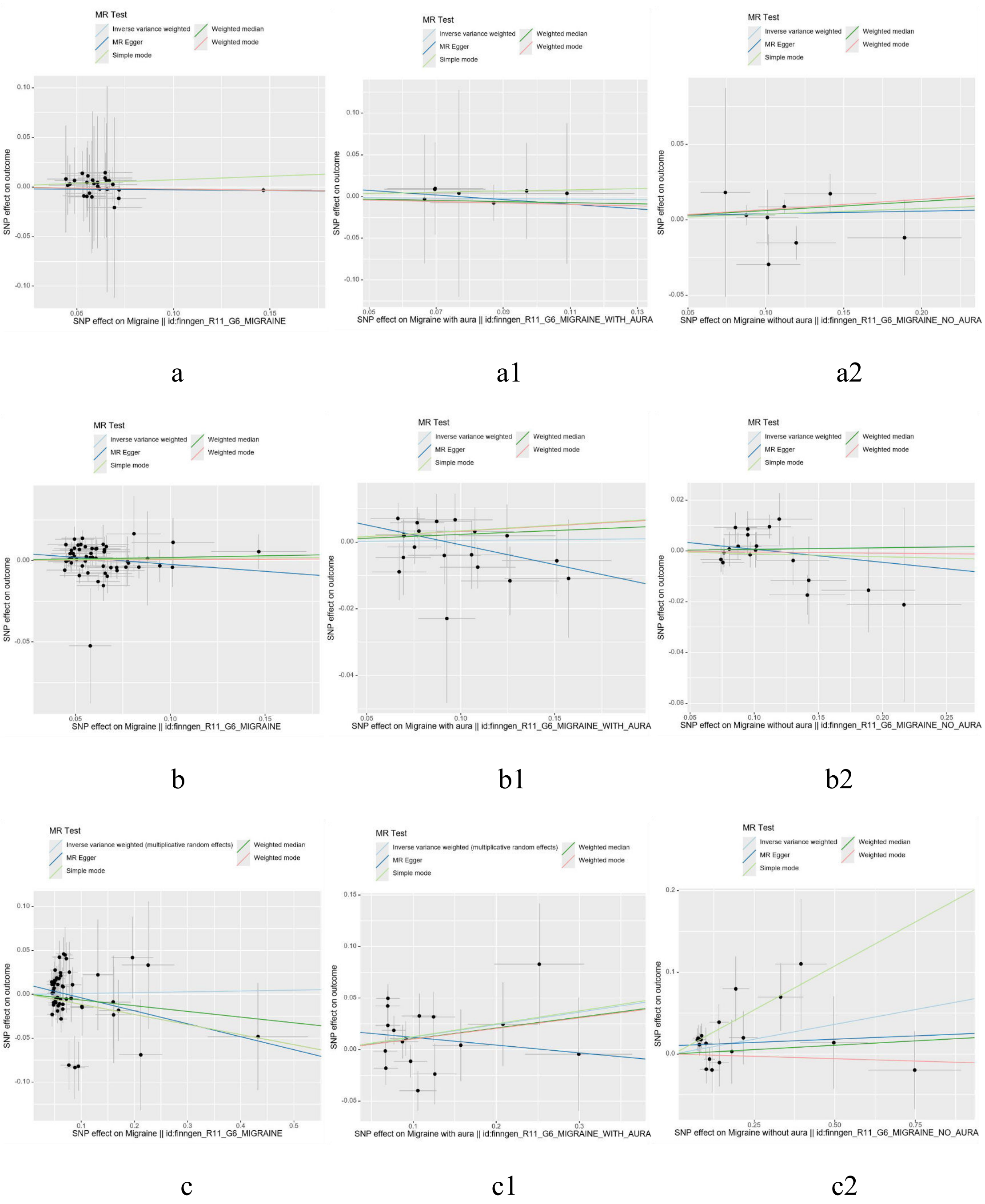

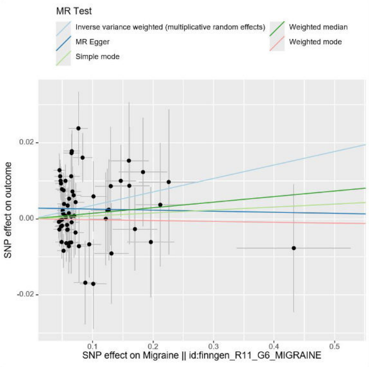

d

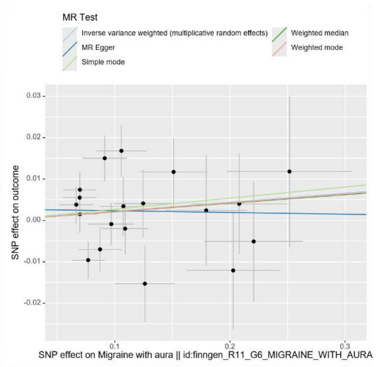

d1

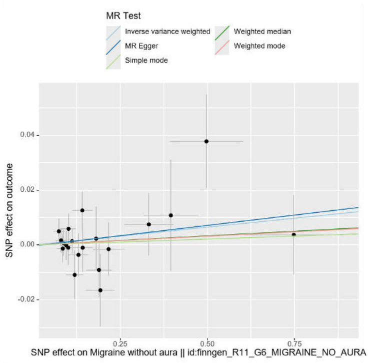

d2

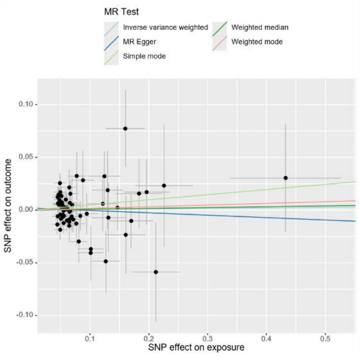

e

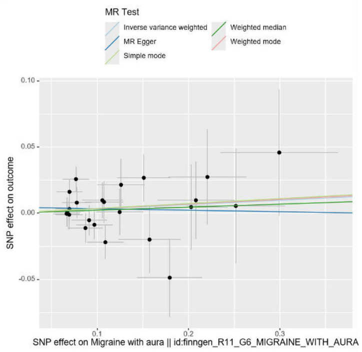

e1

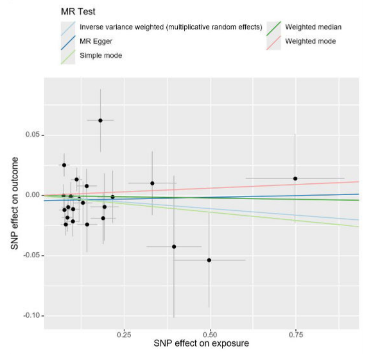

e2

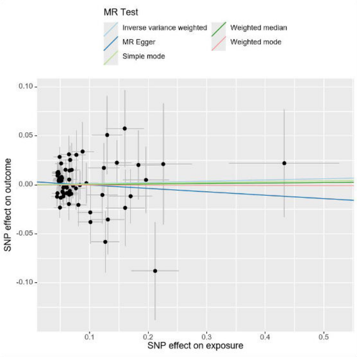

f

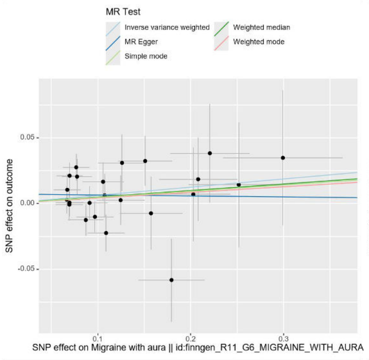

f1

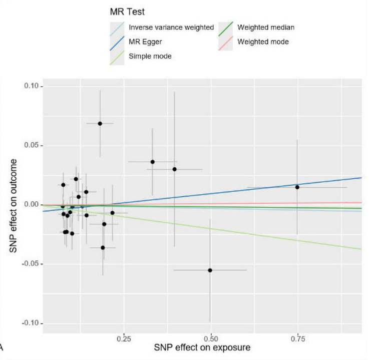

f2

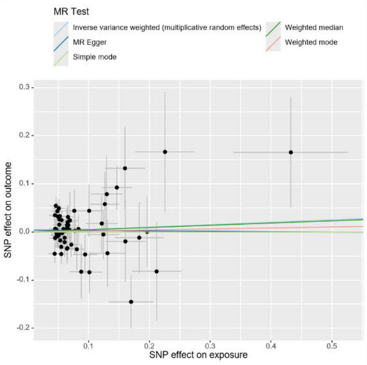

g

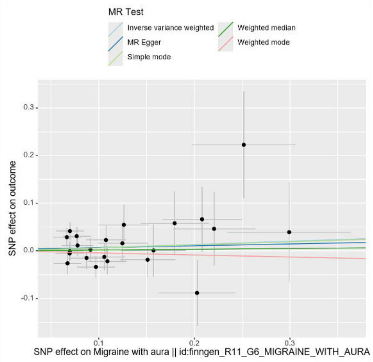

g1

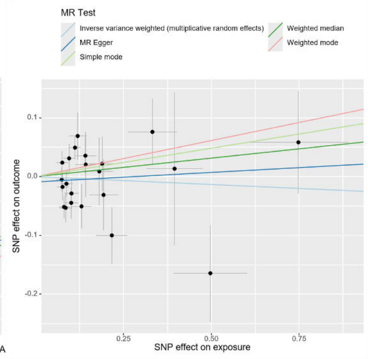

g2

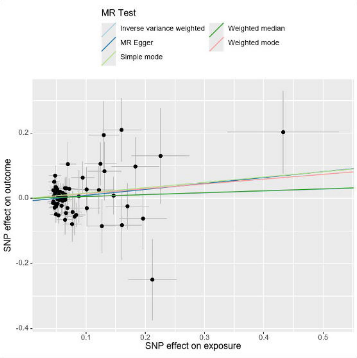

h

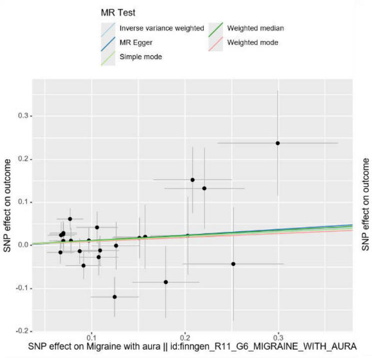

h1

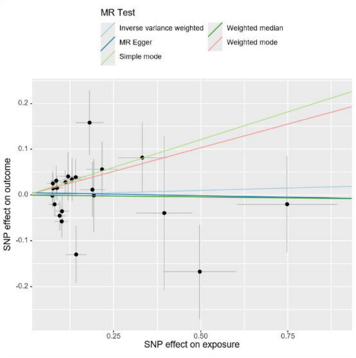

h2

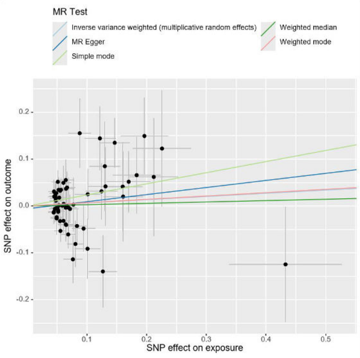

i

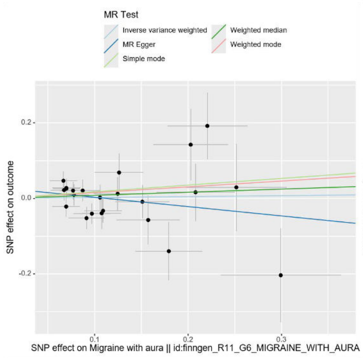

i1

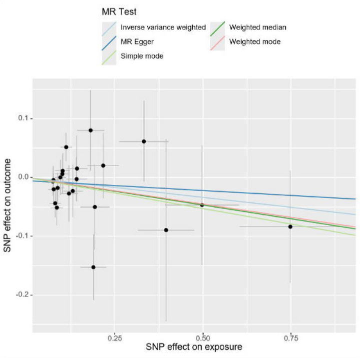

i2

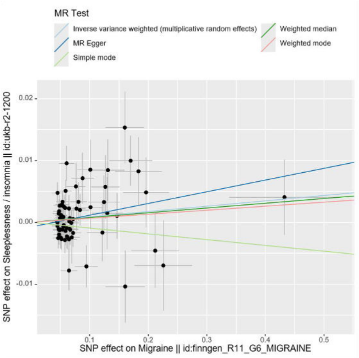

j

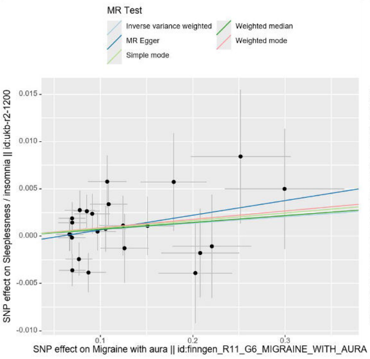

j1

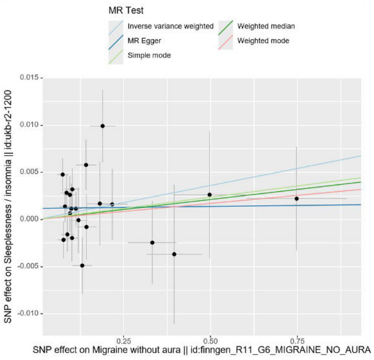

j2

Supplementary Figure 1: Scatter plot of SNPs

a: the scatter plot of the analysis between migraine and epilepsy;

a1: the scatter plot of the analysis between MA and epilepsy;

a2: the scatter plot of the analysis between MO and epilepsy;

b: the scatter plot of the analysis between migraine and anxiety disorder;

b1: the scatter plot of the analysis between MA and anxiety disorder;

b2: the scatter plot of the analysis between MO and anxiety disorder;

c: the scatter plot of the analysis between migraine and bipolar disorder;

c1: the scatter plot of the analysis between MA and bipolar disorder;

c2: the scatter plot of the analysis between MO and bipolar disorder;

d: the scatter plot of the analysis between migraine and major depression;

d1: the scatter plot of the analysis between MA and major depression;

d2: the scatter plot of the analysis between MO and major depression;

e: the scatter plot of the analysis between migraine and AS;

e1: the scatter plot of the analysis between MA and AS;

e2: the scatter plot of the analysis between MO and AS;

f: the scatter plot of the analysis between migraine and AIS;

f1: the scatter plot of the analysis between MA and AIS;

f2: the scatter plot of the analysis between MO and AIS;

g: the scatter plot of the analysis between migraine and CES;

g1: the scatter plot of the analysis between MA and CES;

g2: the scatter plot of the analysis between MO and CES;

h: the scatter plot of the analysis between migraine and LAS;

h1: the scatter plot of the analysis between MA and LAS;

h2: the scatter plot of the analysis between MO and LAS;

i: the scatter plot of the analysis between migraine and SVS;

i1: the scatter plot of the analysis between MA and SVS;

i2: the scatter plot of the analysis between MO and SVS;

j: the scatter plot of the analysis between migraine and insomnia;

j1: the scatter plot of the analysis between MA and insomnia;

j2: the scatter plot of the analysis between MO and insomnia;

Supplementary Figure 2:

Funnel plot

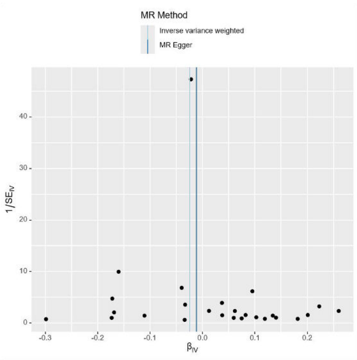

a

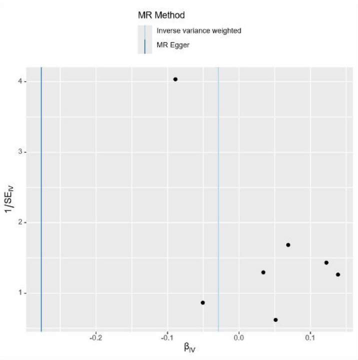

a1

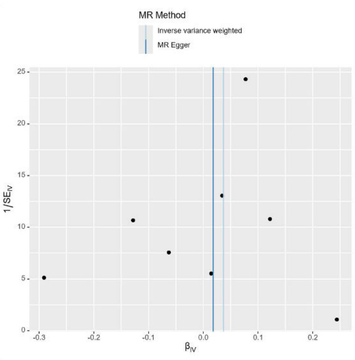

a2

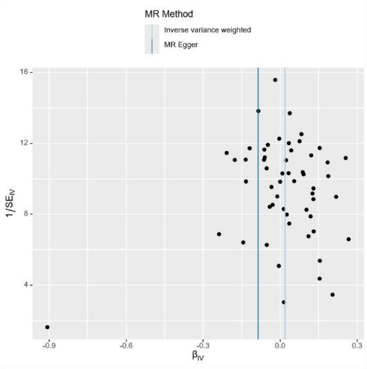

b

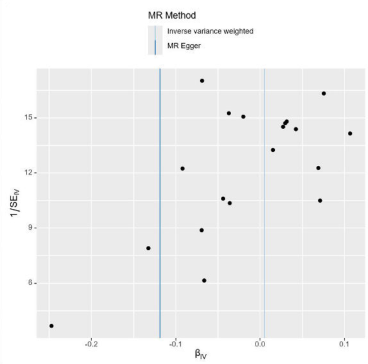

b2

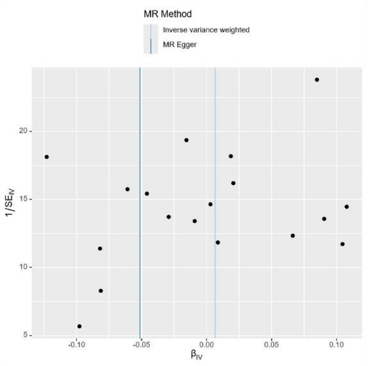

b3

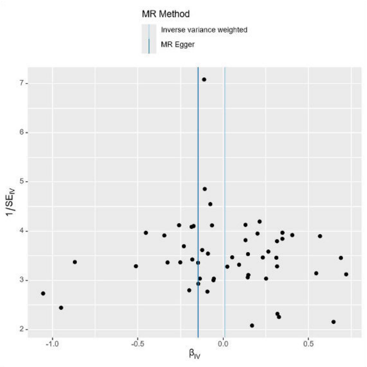

c

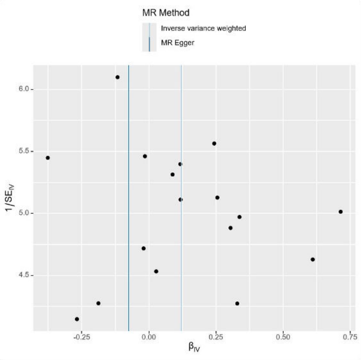

c1

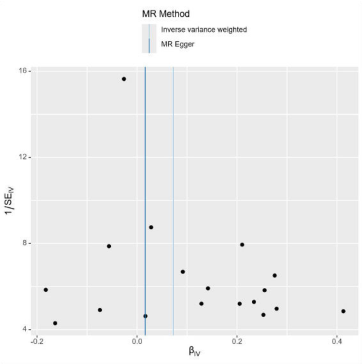

c2

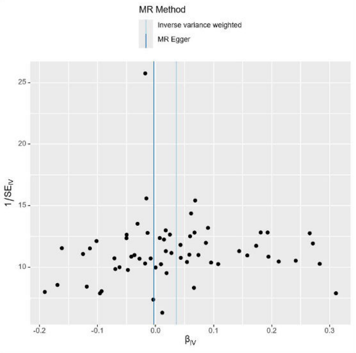

d

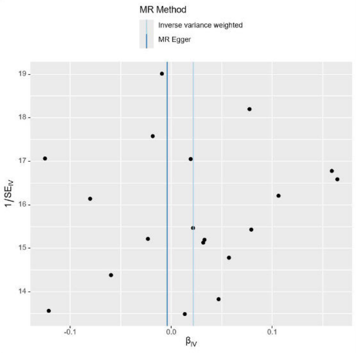

d1

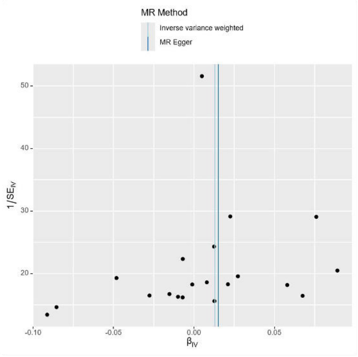

d2

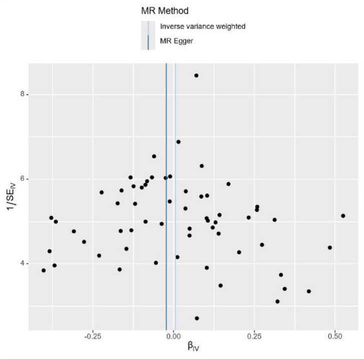

e

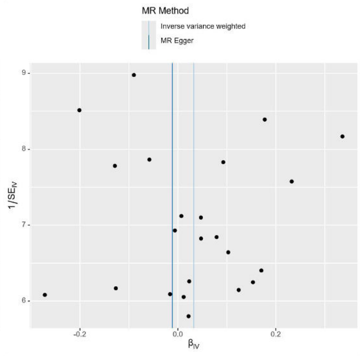

e1

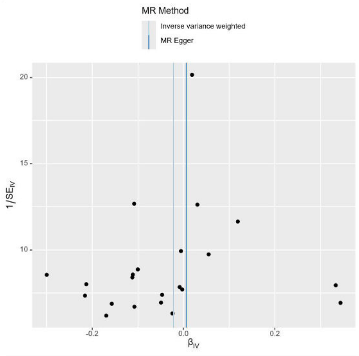

e2

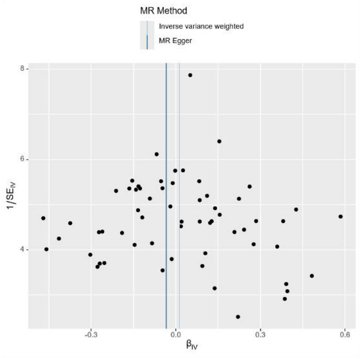

f

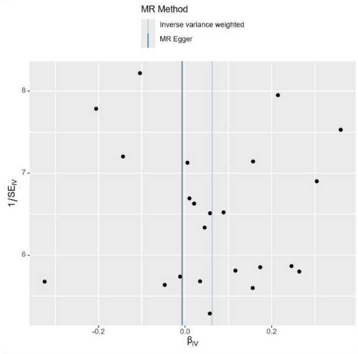

f1

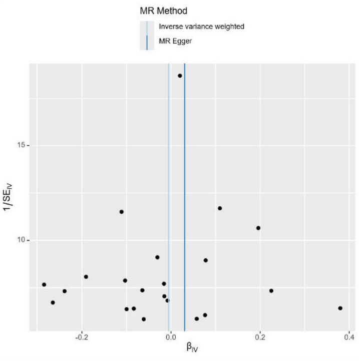

f2

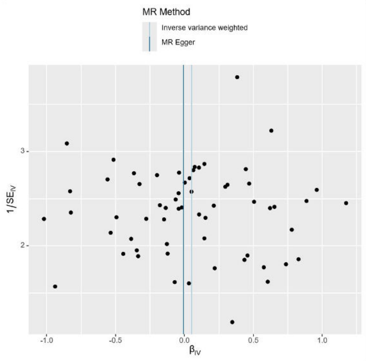

g

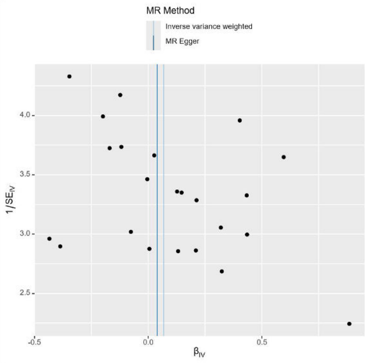

g1

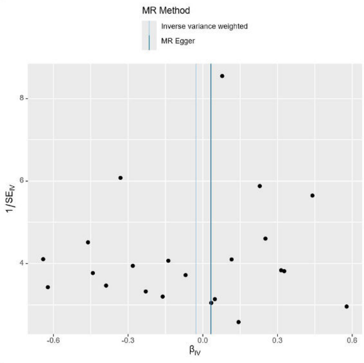

g2

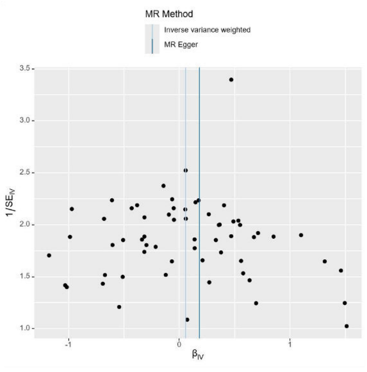

h

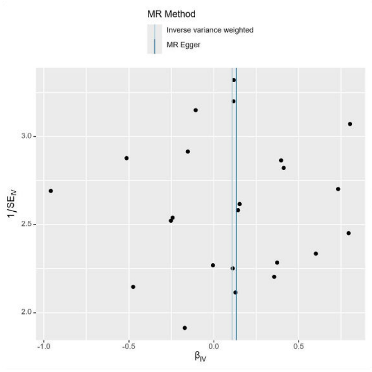

h1

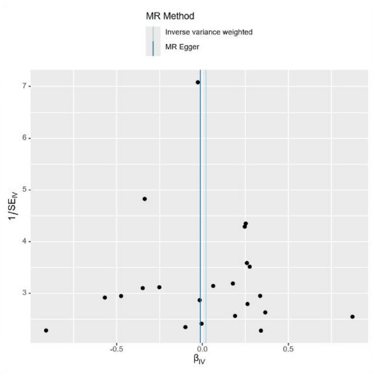

h2

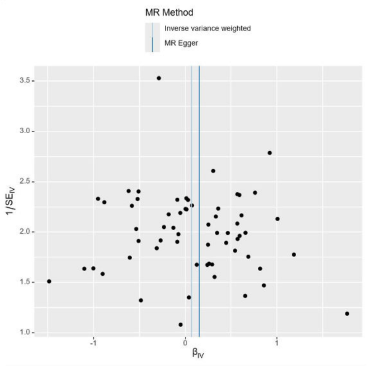

i

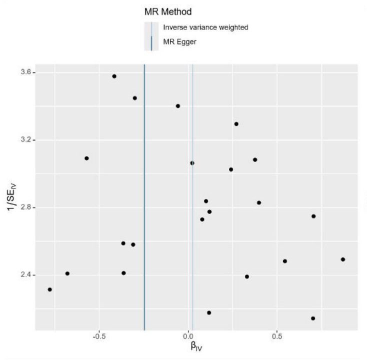

i1

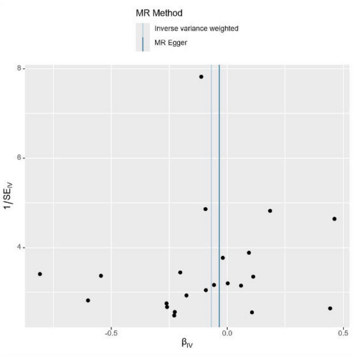

i2

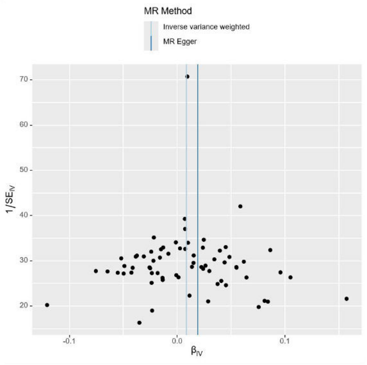

j

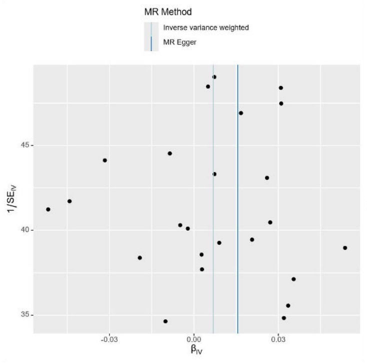

j1

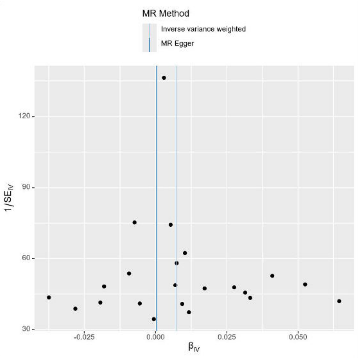

j2

Supplementary Figure 2: Funnel plot of SNPs,

a: the funnel plot of the analysis between migraine and epilepsy;

a1: the funnel plot of the analysis between MA and epilepsy;

a2: the funnel plot of the analysis between MO and epilepsy;

b: the funnel plot of the analysis between migraine and anxiety disorder;

b1: the funnel plot of the analysis between MA and anxiety disorder;

b2: the funnel plot of the analysis between MO and anxiety disorder;

c: the funnel plot of the analysis between migraine and bipolar disorder;

c1: the funnel plot of the analysis between MA and bipolar disorder;

c2: the funnel plot of the analysis between MO and bipolar disorder;

d: the funnel plot of the analysis between migraine and major depression;

d1: the funnel plot of the analysis between MA and major depression;

d2: the funnel plot of the analysis between MO and major depression;

e: the funnel plot of the analysis between migraine and AS;

e1: the funnel plot of the analysis between MA and AS;

e2: the funnel plot of the analysis between MO and AS;

f: the funnel plot of the analysis between migraine and AIS;

f1: the funnel plot of the analysis between MA and AIS;

f2: the funnel plot of the analysis between MO and AIS;

g: the funnel plot of the analysis between migraine and CES;

g1: the funnel plot of the analysis between MA and CES;

g2: the funnel plot of the analysis between MO and CES;

h: the funnel plot of the analysis between migraine and LAS;

h1: the funnel plot of the analysis between MA and LAS;

h2: the funnel plot of the analysis between MO and LAS;

i: the funnel plot of the analysis between migraine and SVS;

i1: the funnel plot of the analysis between MA and SVS;

i2: the funnel plot of the analysis between MO and SVS;

j: the funnel plot of the analysis between migraine and insomnia;

j1: the funnel plot of the analysis between MA and insomnia;

j2: the funnel plot of the analysis between MO and insomnia;

Supplementary Figure 3:

Leave-one-out plot

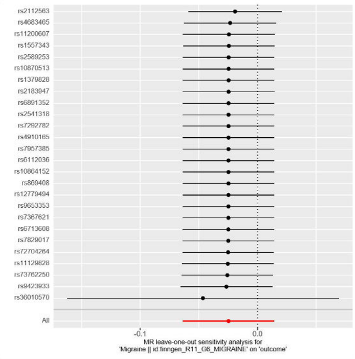
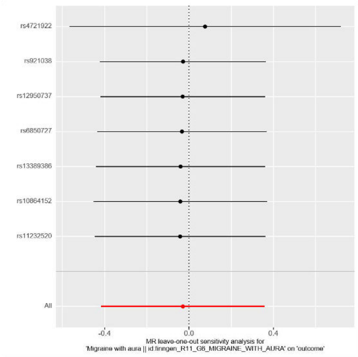
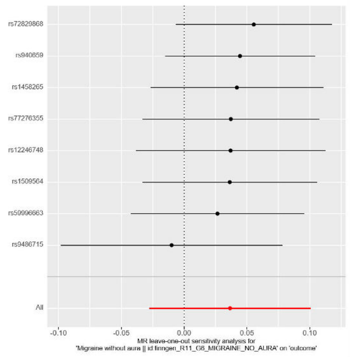

a a1 a2

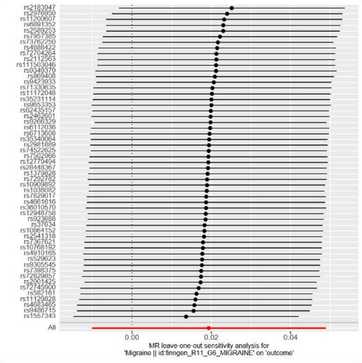
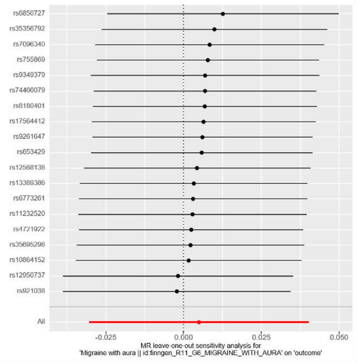
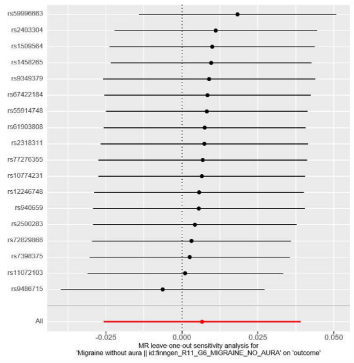

b b1 b2

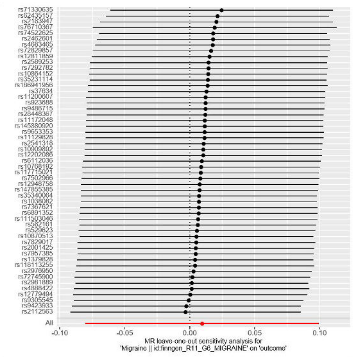
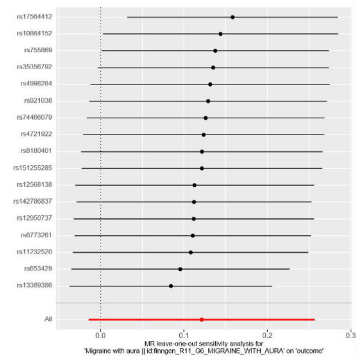
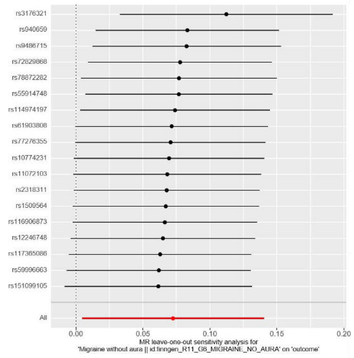

c c1 c2

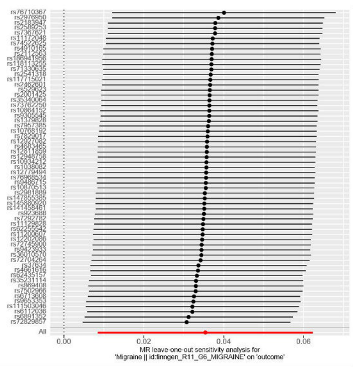

d

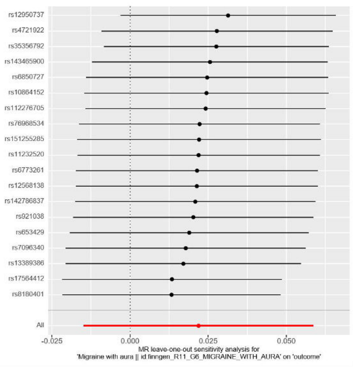

d1

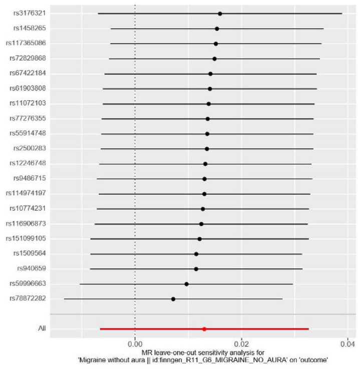

d2

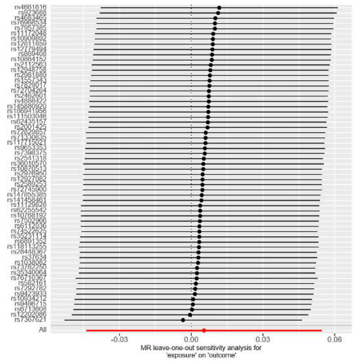

e

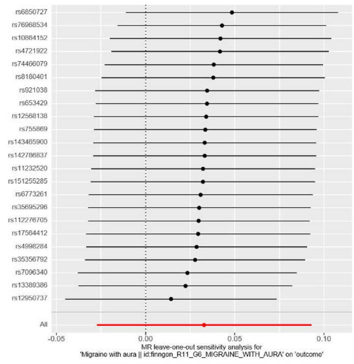

e1

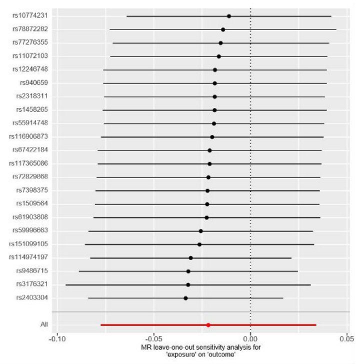

e2

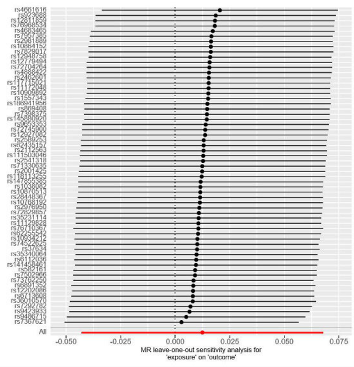

f

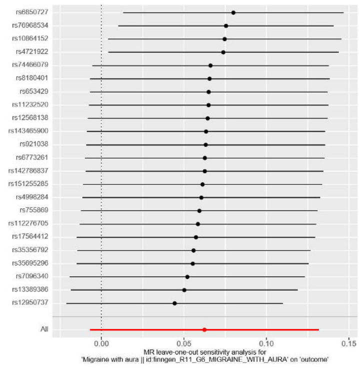

f1

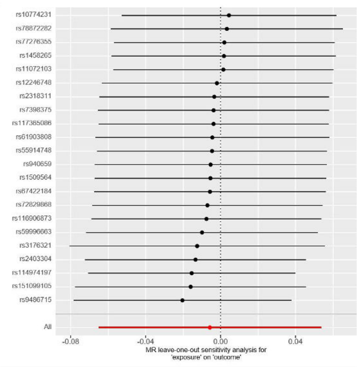

f2

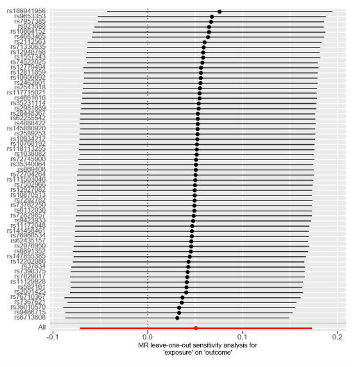

g

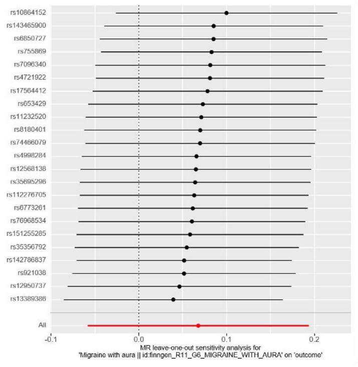

g1

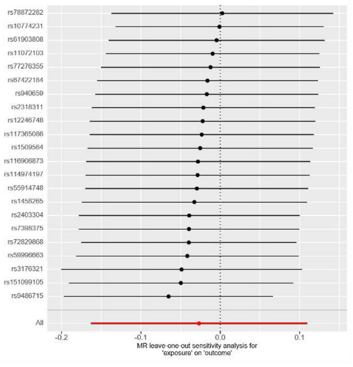

g2

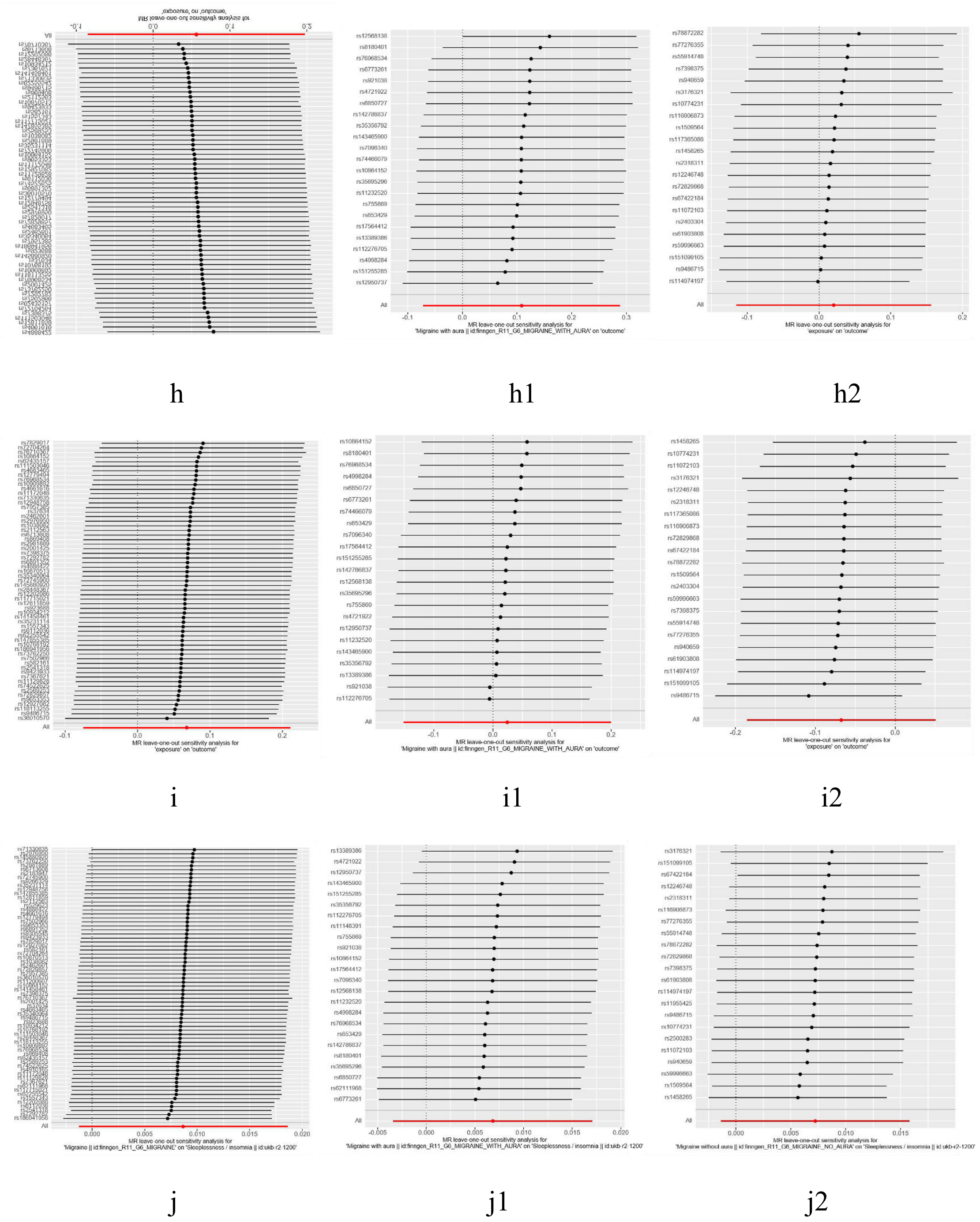

Supplementary Figure 3: Leave-one-out plot of SNPs,

a: the leave-one-out plot of the analysis between migraine and epilepsy;

a1: the leave-one-out plot of the analysis between MA and epilepsy;

a2: the leave-one-out plot of the analysis between MO and epilepsy;

b: the leave-one-out plot of the analysis between migraine and anxiety disorder;

b1: the leave-one-out plot of the analysis between MA and anxiety disorder;

b2: the leave-one-out plot of the analysis between MO and anxiety disorder;

c: the leave-one-out plot of the analysis between migraine and bipolar disorder;

c1: the leave-one-out plot of the analysis between MA and bipolar disorder;

c2: the leave-one-out plot of the analysis between MO and bipolar disorder;

d: the leave-one-out plot of the analysis between migraine and major depression;

d1: the leave-one-out plot of the analysis between MA and major depression;

d2: the leave-one-out plot of the analysis between MO and major depression;

e: the leave-one-out plot of the analysis between migraine and AS;

e1: the leave-one-out plot of the analysis between MA and AS;

e2: the leave-one-out plot of the analysis between MO and AS;

f: the leave-one-out plot of the analysis between migraine and AIS;

f1: the leave-one-out plot of the analysis between MA and AIS;

f2: the leave-one-out plot of the analysis between MO and AIS;

g: the leave-one-out plot of the analysis between migraine and CES;

g1: the leave-one-out plot of the analysis between MA and CES;

g2: the leave-one-out plot of the analysis between MO and CES;

h: the leave-one-out plot of the analysis between migraine and LAS;

h1: the leave-one-out plot of the analysis between MA and LAS;

h2: the leave-one-out plot of the analysis between MO and LAS;

i: the leave-one-out plot of the analysis between migraine and SVS;

i1: the leave-one-out plot of the analysis between MA and SVS;

i2: the leave-one-out plot of the analysis between MO and SVS;

j: the leave-one-out plot of the analysis between migraine and insomnia;

j1: the leave-one-out plot of the analysis between MA and insomnia;

j2: the leave-one-out plot of the analysis between MO and insomnia;

Supplementary Figure 4:

Forest plot

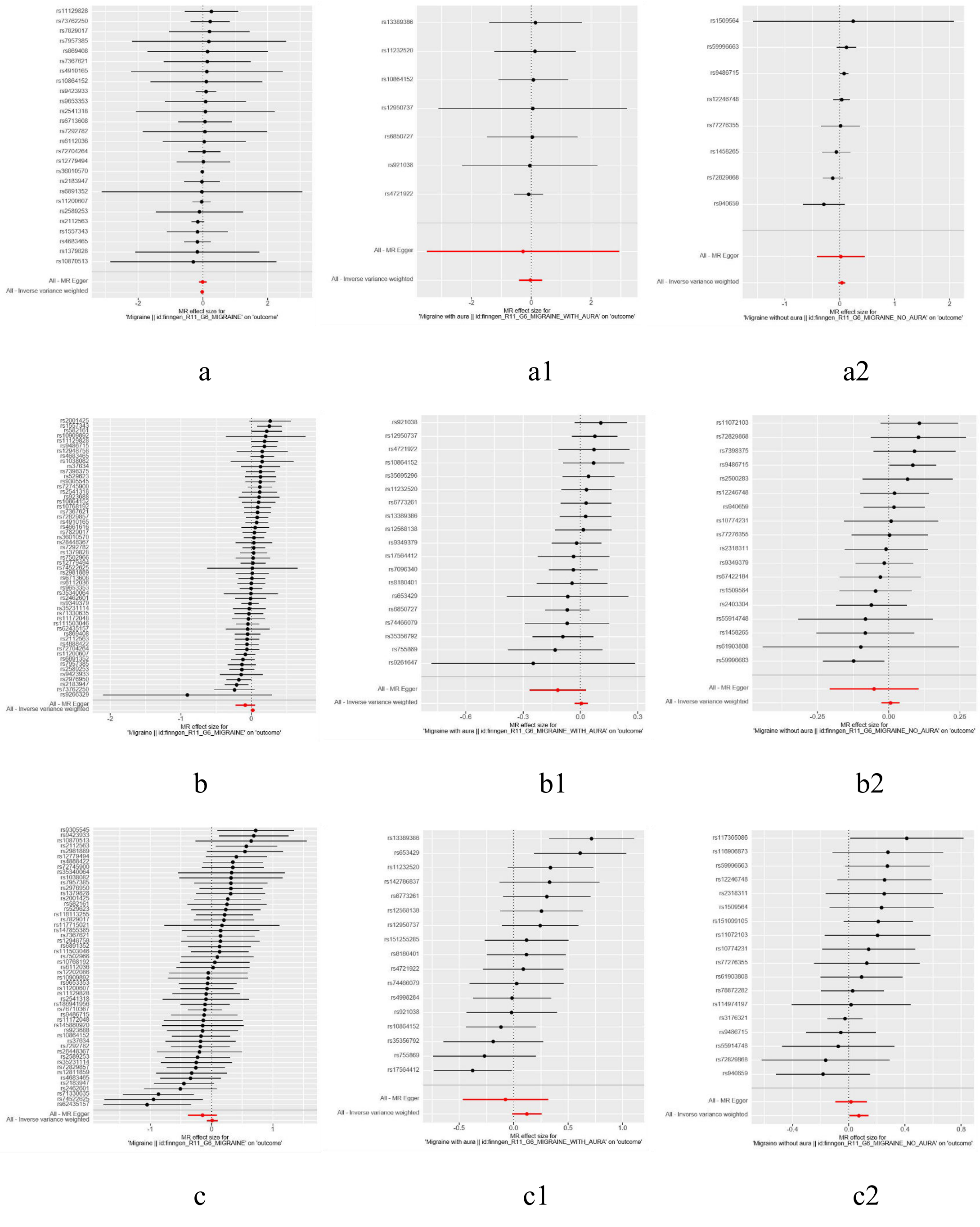

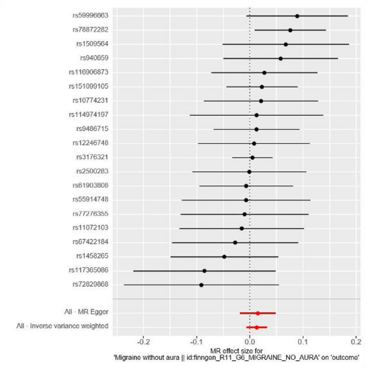

d

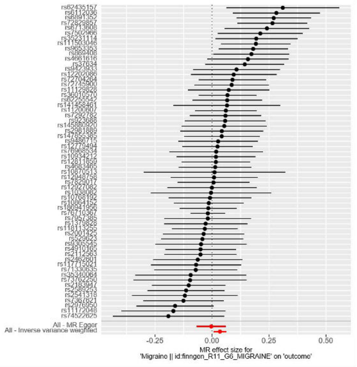

d1

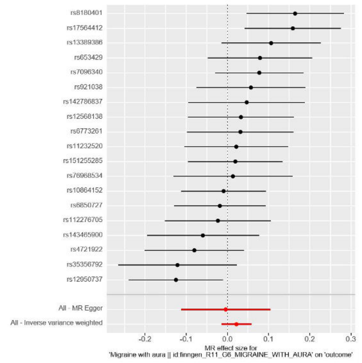

d2

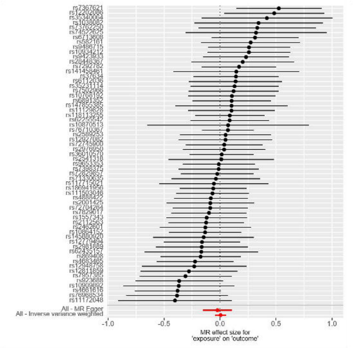

e

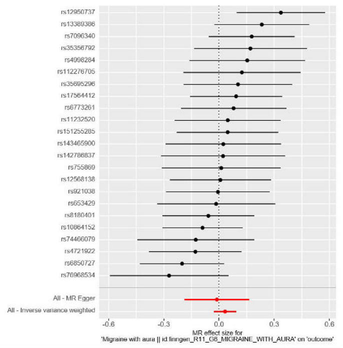

e1

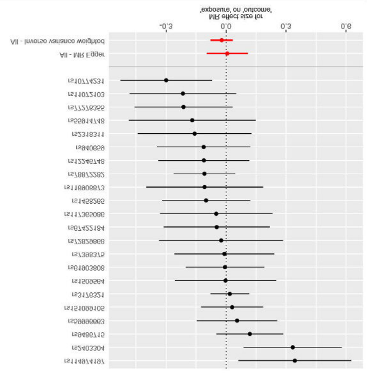

e2

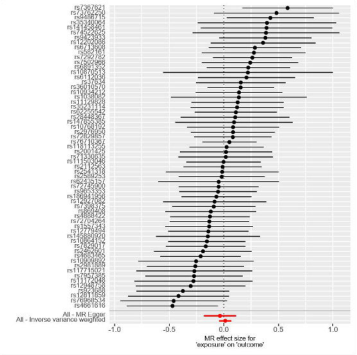

f

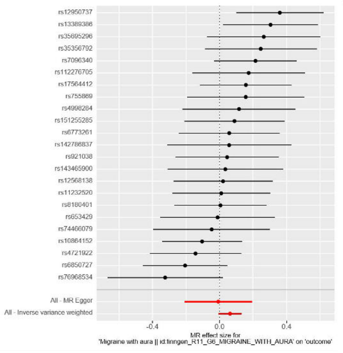

f1

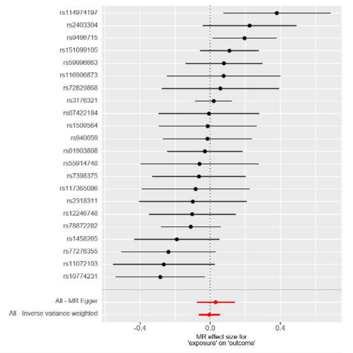

f2

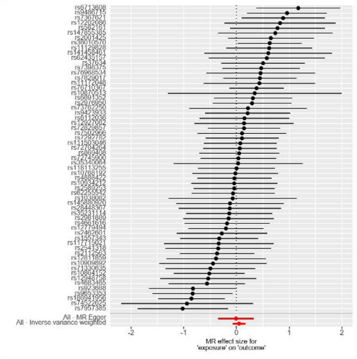

g

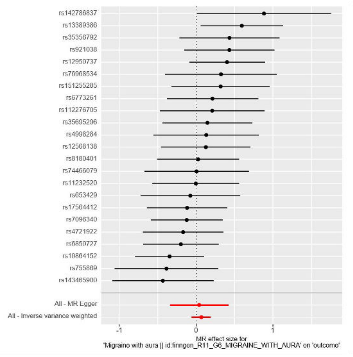

g1

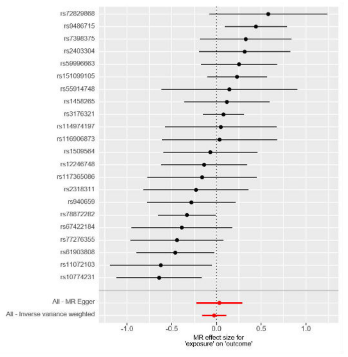

g2

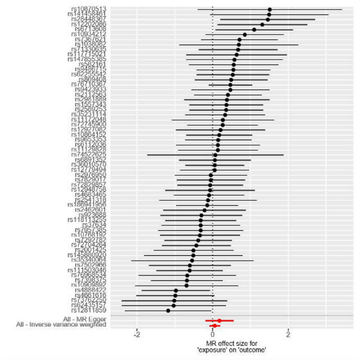

h

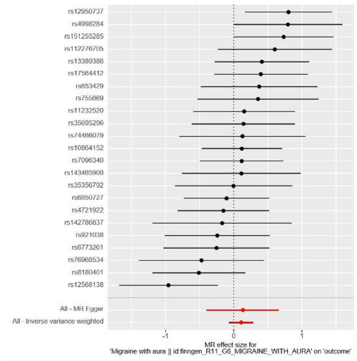

h1

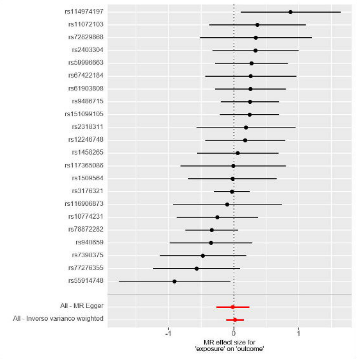

h2

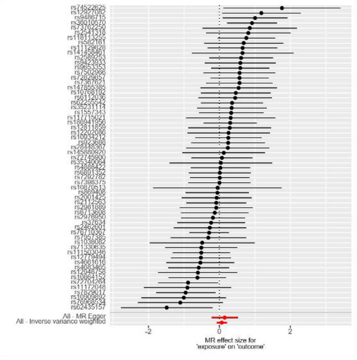

i

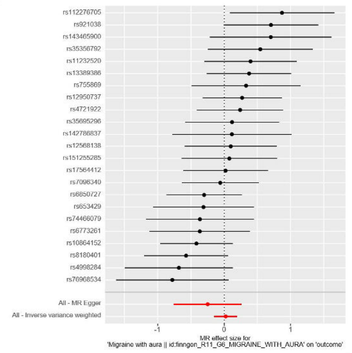

i1

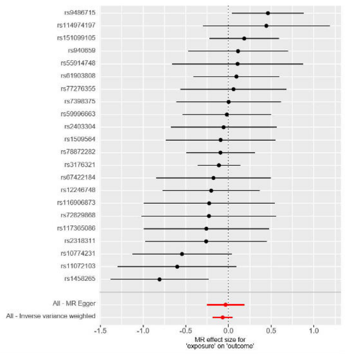

i2

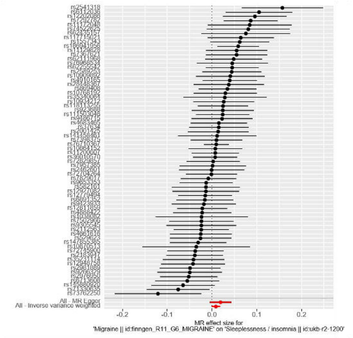

j

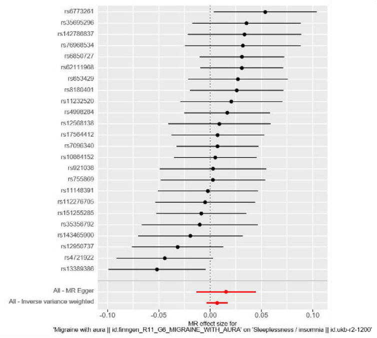

j1

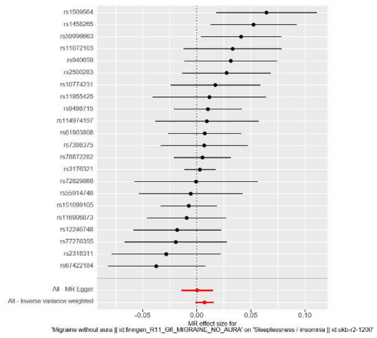

j2

a: forest plot of the causal effect of migraine with epilepsy and associated SNPs;

a1: forest plot of the causal effect of MA with epilepsy and associated SNPs;

a2: forest plot of the causal effect of MO with epilepsy and associated SNPs;

b: forest plot of the causal effect of migraine with anxiety disorder and associated SNPs;

b1: forest plot of the causal effect of MA with anxiety disorder and associated SNPs;

b2: forest plot of the causal effect of MO with anxiety disorder and associated SNPs;

c: forest plot of the causal effect of migraine with bipolar disorder and associated SNPs;

c1: forest plot of the causal effect of MA with bipolar disorder and associated SNPs;

c2: forest plot of the causal effect of MO with bipolar disorder and associated SNPs;

d: forest plot of the causal effect of migraine with major depressive disorder and associated SNPs;

d1: forest plot of the causal effect of MA with major depressive disorder and associated SNPs;

d2: forest plot of the causal effect of MO with major depressive disorder and associated SNPs;

e: forest plot of the causal effect of migraine with AS and associated SNPs;

e1: forest plot of the causal effect of MA with AS and associated SNPs;

e2: forest plot of the causal effect of MO with AS and associated SNPs;

f: forest plot of the causal effect of migraine with AIS and associated SNPs;

f1: forest plot of the causal effect of MA with AIS and associated SNPs;

f2: forest plot of the causal effect of MO with AIS and associated SNPs;

g: forest plot of the causal effect of migraine with CES and associated SNPs;

g1: forest plot of the causal effect of MA with CES and associated SNPs;

g2: forest plot of the causal effect of MO with CES and associated SNPs;

h: forest plot of the causal effect of migraine with LAS and associated SNPs;

h1: forest plot of the causal effect of MA with LAS and associated SNPs;

h2: forest plot of the causal effect of MO with LAS and associated SNPs;

i: forest plot of the causal effect of migraine with SVS and associated SNPs;

i1: forest plot of the causal effect of MA with SVS and associated SNPs;

i2: forest plot of the causal effect of MO with SVS and associated SNPs;

j: forest plot of the causal effect of migraine with insomnia and associated SNPs;

j1: forest plot of the causal effect of MA with insomnia and associated SNPs;

j2: forest plot of the causal effect of MO with insomnia and associated SNPs;
